# Different roles of host and habitat in determining the microbial communities of plant-feeding true bugs

**DOI:** 10.1186/s40168-023-01702-y

**Published:** 2023-11-07

**Authors:** Zi-Wen Yang, Jiu-Yang Luo, Yu Men, Zhi-Hui Liu, Zi-Kai Zheng, Yan-Hui Wang, Qiang Xie

**Affiliations:** 1 School of Life Sciences, State Key Laboratory of Biocontrol, Sun Yat-sen University, Guangzhou, 510275 Guangdong China; 2 School of Life Sciences, Zhaoqing University, Zhaoqing, 526061 China

**Keywords:** Phytophagous true bugs, Amplicon sequencing, Symbiotic bacteria, Symbiotic fungi, Microbial community, Third-generation sequencing

## Abstract

**Background:**

The true bugs (Heteroptera) occupy nearly all of the known ecological niches of insects. Among them, as a group containing more than 30,000 species, the phytophagous true bugs are making increasing impacts on agricultural and forestry ecosystems. Previous studies proved that symbiotic bacteria play important roles in these insects in fitting various habitats. However, it is still obscure about the evolutionary and ecological patterns of the microorganisms of phytophagous true bugs as a whole with comprehensive taxon sampling.

**Results:**

Here, in order to explore the symbiotic patterns between plant-feeding true bugs and their symbiotic microorganisms, 209 species belonging to 32 families of 9 superfamilies had been sampled, which covered all the major phytophagous families of true bugs. The symbiotic microbial communities were surveyed by full-length 16S rRNA gene and ITS amplicons respectively for bacteria and fungi using the PacBio platform. We revealed that hosts mainly affect the dominant bacteria of symbiotic microbial communities, while habitats generally influence the subordinate ones. Thereafter, we carried out the ancestral state reconstruction of the dominant bacteria and found that dramatic replacements of dominant bacteria occurred in the early Cretaceous and formed newly stable symbiotic relationships accompanying the radiation of insect families. In contrast, the symbiotic fungi were revealed to be horizontally transmitted, which makes fungal communities distinctive in different habitats but not significantly related to hosts.

**Conclusions:**

Host and habitat determine microbial communities of plant-feeding true bugs in different roles. The symbiotic bacterial communities are both shaped by host and habitat but in different ways. Nevertheless, the symbiotic fungal communities are mainly influenced by habitat but not host. These findings shed light on a general framework for future microbiome research of phytophagous insects.

Video Abstract

**Supplementary Information:**

The online version contains supplementary material available at 10.1186/s40168-023-01702-y.

## Introduction

Most of the plant-feeding true bugs belong to five superfamilies, including Miroidea, Pentatomoidea, Pyrrhocoroidea, Coreoidea, and Lygaeoidea [1–3], among which Miroidea belongs to the infraorder Cimicomorpha while the other four belong to Pentatomomorpha. More than 30,000 species are contained in these superfamilies and thus comprise nearly two-thirds of the species diversity of true bugs [4]. Phytophagous true bugs are also highly diversified in host plants and ecological niches [3, 5]. Many important agriculture pests such as *Lygus lucorum* (Miroidea: Miridae), *Halyomorpha halys* (Pentatomoidea: Pentatomidae), and *Riptortus pedestris* (Coreoidea: Alydidae) belong to these groups [6–8].

The established symbiotic relationships with microorganisms contribute to the diversification of ecological niches and species in various insects. Recent studies find that symbionts can confer important physiological traits to their hosts such as digestion, nutrition, and defense allowing insects to adopt new lifestyles and to colonize niches otherwise inaccessible [9–17]. As for the phytophagous true bug superfamilies, Miroidea is the largest group known to have complex feeding habits, ranging from strictly phytophagous, omnivorous, to strictly carnivorous [18]. Symbiotic bacteria are important to mirid's nutrition and survival while feeding on plants or prey [19, 20]. The stink bugs (Pentatomoidea) are generally symbiotic with *Pantoea*, which can neither develop nor reproduce normally without this kind of bacteria [21–23]. Besides, the genus *Caballeronia*, which has been reclassified from *Burkholderia* in recent years, can not only provide essential amino acids and B vitamins but also stimulate fertility to the severe bean pest *Riptortus pedestris* belonging to Coreoidea [24–26].

Owing to the importance of symbiotic microorganisms, phytophagous true bugs have generally evolved special organs or complex gut constructions to harbor these symbionts. In Lygaeoidea, many species harbor symbionts by a particular organ called bacteriome [27], which is commonly regarded as existing in aphids, psyllids or whiteflies [28]. In contrast, insects of Pentatomoidea and their related groups lack this kind of organ but harbor symbiotic bacteria in the midgut M4 region called crypts instead [29–32]. Whereas, in Pyrrhocoridae, the midgut M4 region is absent and the symbionts are stored in the midgut M3 region [33]. In order to acquire these symbionts, newborns have evolved different ways to realize this goal, including horizontal and vertical transmission modes [29, 30, 32–35]. Under such tight symbiotic relationships, some plant-feeding true bugs and their symbiotic bacteria have been found to coevolve phylogenetically [36, 37]. However, it is still short of recognition of the symbiotic relationship between phytophagous true bugs and their symbiotic bacteria with comprehensive taxon sampling. Besides, the symbiotic fungi, which play important roles in other insects, are seldom reported in the previous research on true bugs making us know little about their community structure and symbiotic pattern.

In this study, we took an in-depth look at the symbiotic pattern of both symbiotic bacteria and fungi in the plant-feeding true bugs and their closely related groups. In order to solve the problems mentioned above, over 200 species in 32 families representing all major phytophagous true bugs at the family level were sampled (Supplementary Figure S1, Supplementary Table S1). With the contribution of complete taxon sampling and full-length 16S rRNA gene and ITS amplicon sequencing, our study addresses the general patterns of evolutionary and ecological factors influencing the symbiotic microbial communities of phytophagous true bugs.

## Results

### Symbiotic microbial community overall

The bacterial and fungal communities were respectively characterized by full-length 16S rRNA gene and ITS using the PacBio Sequel II platform, which yielded a total of 1,003,498 and 1,105,734 sequences. After denoising and filtering, 3723 bacterial amplicon sequence variants (ASVs) and 3 195 fungal ASVs had been detected. The bacteria phylum *Proteobacteria* (87.97%) took the largest proportion in most samples (Supplementary Figure S2a, c, e), and meanwhile, the fungal phylum *Ascomycota* (58.02%) was the most abundant fungi and *Basidiomycota* (24.90%) took a high proportion in some humid species samples (Supplementary Figure S2b, d, f).

**Fig. 1:**
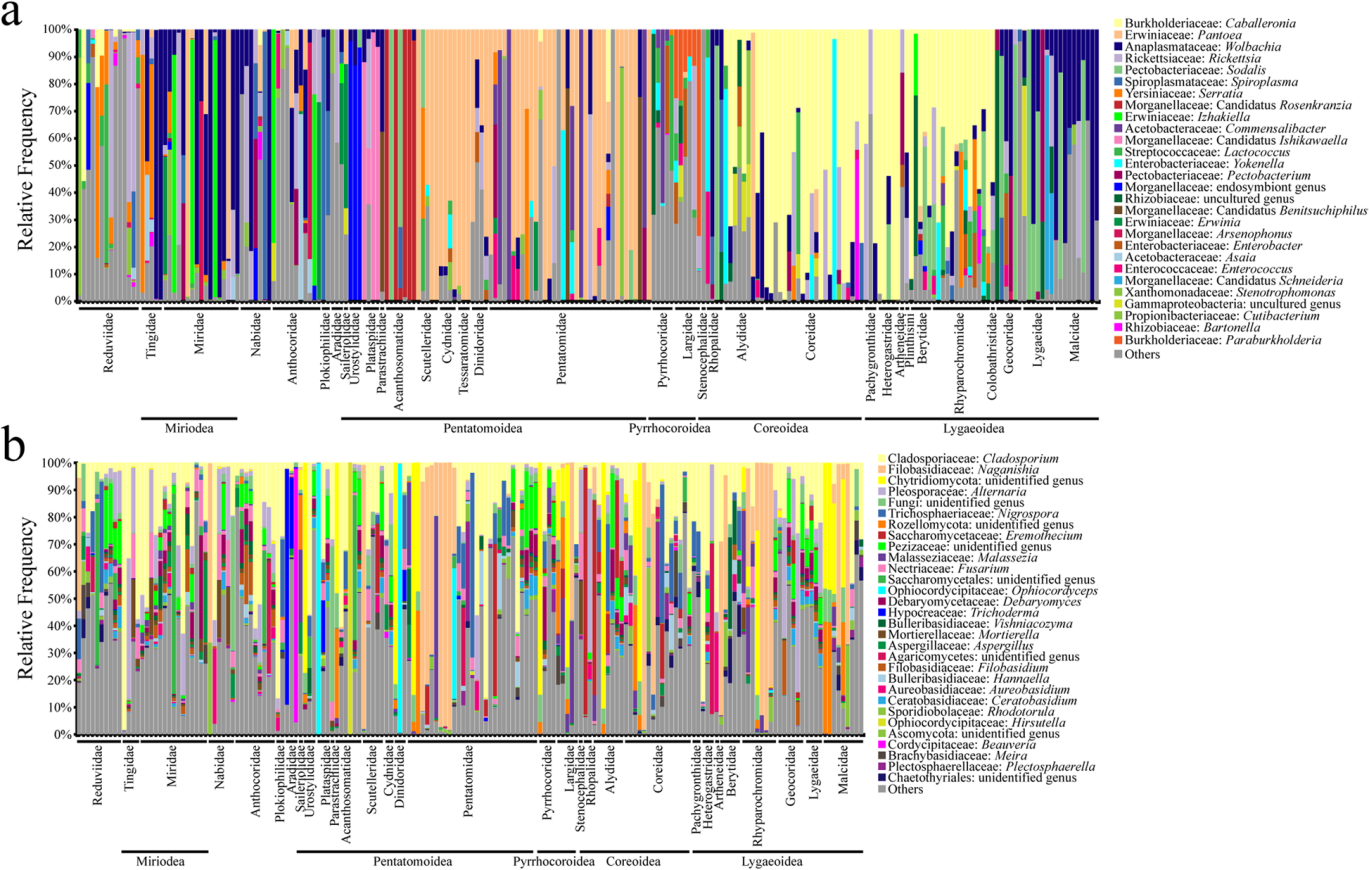
Composition of symbiotic bacterial and fungal communities in plant-feeding true bugs. The relative abundance plots of bacteria (**a**, *n* = 225) and fungi (**b**, *n* = 174) are displayed. Totally 28 genera in bacterial communities and 29 genera in fungal communities are shown, which represent the most abundant genera. The remaining genera are summarized as others. In cases where there is no genus-level identification based on the Silva taxonomy, the family-level classification is also given. The samples are grouped according to the hosts and are listed according to their phylogenetic relationship. Besides, the five phytophagous true bug superfamilies are shown at the bottom of the plots

In the phytophagous true bugs, *Wolbachia* took the largest number of bacteria in the superfamily Miroidea. The bacterial communities of Pentatomoidea were dominated by the genera of *Erwiniaceae* and *Morganellaceae*. The genera of *Burkholderiaceae* like *Caballeronia* took the largest proportion in Pyrrhocoroidea, Coreoidea, and Lygaeoidea. Nevertheless, the family Rhopalidae belonging to Coreoidea lacked *Caballeronia* and no other bacteria genus had been shown as the dominant one. In the other true bugs, no bacteria genus could be found occupying the vast majority in Reduvioidea. Two genera *Wolbachia* and *Rickettsia* took the highest proportion of the first investigated family Anthocoridae. As for the other families of Cimicomorpha that had never been studied before this study, the case of Nabidae was similar to that of Anthocoridae, in contrast to Plokiophilidae mainly containing *Spiroplasma* in the symbiotic bacterial community (Fig. 1a).

In the part of fungi, it could be found that *Cladosporium* distributed widely in most samples. The *Naganishia* were also distributed widely but only took a high proportion in some samples from southeast China. Whereas, no obviously major groups had been detected at the family level of fungi (Fig. 1b).

### Factors influencing microbial communities of phytophagous true bugs

**Fig. 2:**
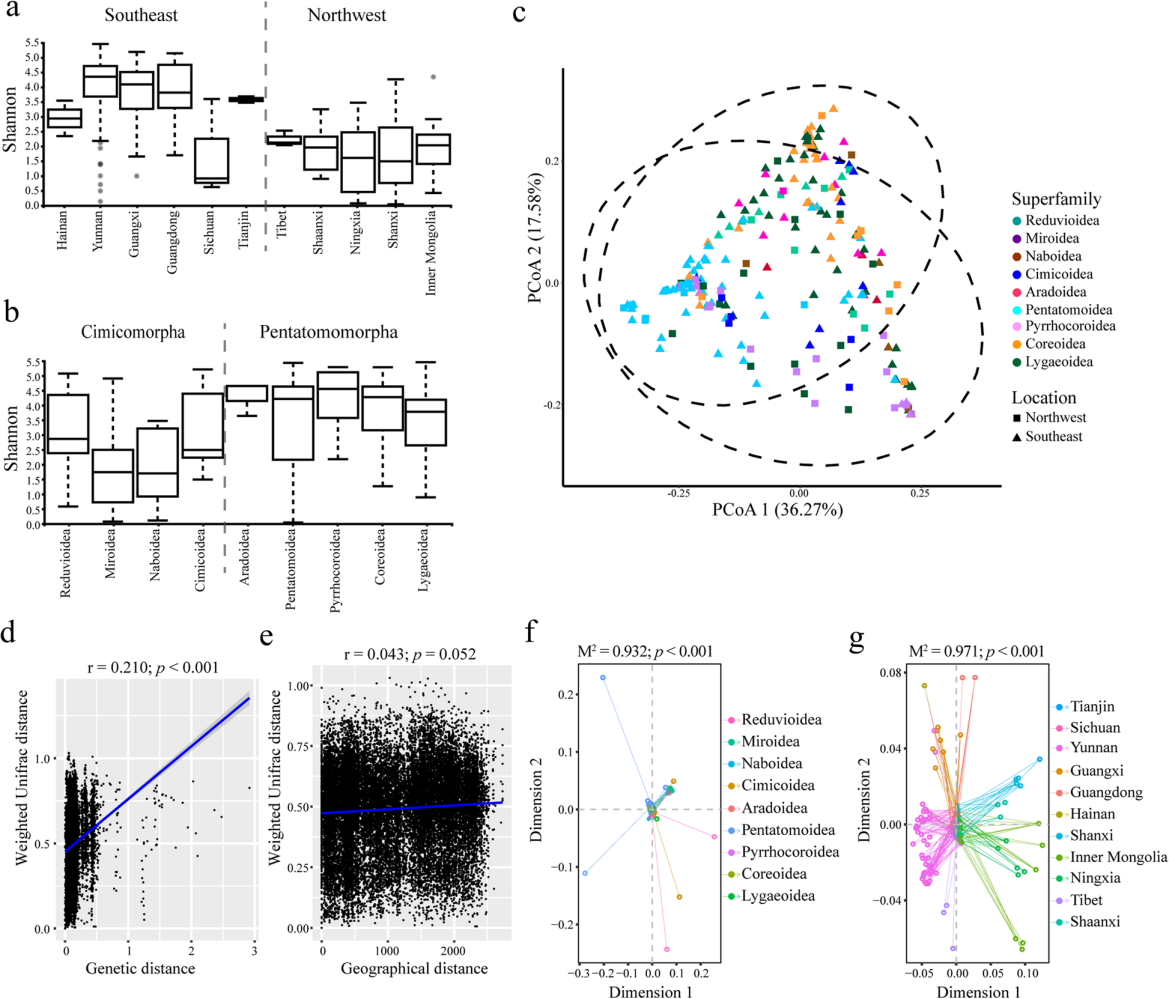
Factors that influence symbiotic bacterial communities. Insect samples collected in the same province of China or belonging to the same superfamily are grouped. The sample locations are divided into southeast and northwest of China according to the Heihe-Tengchong Line. The superfamilies are classified into Cimicomorpha and Pentatomomorpha according to their phylogenetic relationship. The alpha diversity results of symbiotic bacterial communities are shown by the Shannon index (**a**,** b**) and are tested the differences by the Kruskal–Wallis test. The beta diversity results are calculated by the weighted Unifrac distance and displayed using the PCoA plot, the confidence ellipsoids (confidence level = 0.95) of different locations are provided by dotted lines (**c**). The sample groups are marked according to insect superfamilies (colors) or sample locations (geometrical shape). The Mantel test (**d**,** e**) and the Procrustes test (**f**,** g**) are displayed at the bottom half. If *p* < 0.05, it could be regarded that the symbiotic bacterial communities are related to the host genetic distance or the sample geographical distance

Alpha diversity of symbiotic bacteria was concurrently affected by host and environment (Fig. 2a, b, Supplementary Figure S3a–d). The samples belonging to Pentatomomorpha and southeast China had higher Shannon indexes compared with other taxa and locations (*p* < 0.001; Fig. 2a, b, Supplementary Figure S3a). As for the environment, the richness of bacterial communities is significantly influenced by ecological factors like temperature and humidity (*p* < 0.001; Supplementary Figure S3b, c). While, for altitude, the low-relief mountain had the highest indexes but did not show a significant difference with the others (*p* > 0.05; Supplementary Figure S3d).

In the phytophagous superfamilies, the PCoA plots showed that Pentatomoidea had unique symbiotic bacterial communities. The symbiotic bacterial communities of Pyrrhocoroidea, Coreoidea, and Lygaeoidea possessed a certain degree of similarity. Samples of Miroidea had similar bacterial communities to those of Naboidea. Besides, an obvious cluster relationship was not observed between the samples collected in different locations (Fig. 2c). These results were supported by the PERMANOVA test when conducting the statistical analyses by the weighted Unifrac distance matrix (Supplementary Tables S3 and S4). To further verify the results mentioned above, correlations between symbiotic bacterial communities and the distance of the host phylogeny or living regions were examined by the Mantel and Procrustes tests. These tests indicated that symbiotic bacterial communities were significantly correlated with the hosts (*P* < 0.001; Fig. 2d, f). In addition, the bacterial communities did not show statistical relevance to the distance of living regions according to the Mantel test (*p* = 0.052; Fig. 2e). The Procrustes test showed a different result compared with that of the Mantel test in geographic distance, which may be due to the specific regional geographic distribution of insects.

**Fig. 3:**
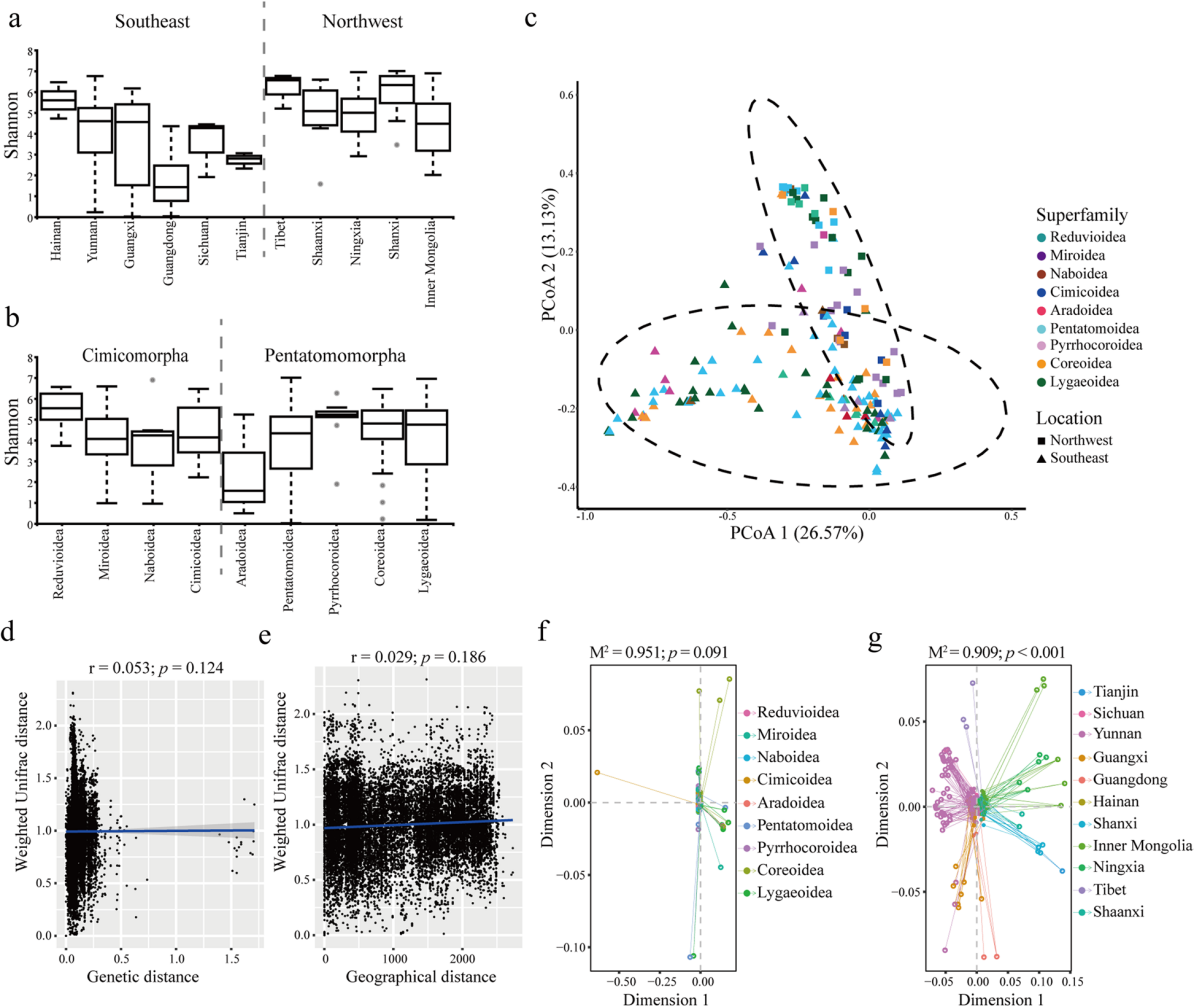
The factors that influence symbiotic fungal communities. The samples grouped in the fungal community analyses are the same as those grouped in bacteria. The alpha diversity results of symbiotic fungal communities are shown by the Shannon index (**a**,** b**) and are tested the differences by the Kruskal–Wallis test. The beta diversity results are calculated using the weighted Unifrac distance and displayed by the PCoA plot, the confidence ellipsoids (confidence level = 0.95) of different locations are provided by dotted lines (**c**). The sample groups are marked according to insect superfamilies (colors) or sample locations (geometrical shape). The Mantel test (**d**,** e**) and the Procrustes test (**f**,** g**) are displayed at the bottom half. If *p* < 0.05, it could be regarded that the symbiotic bacterial communities are related to the host genetic distance or the sample geographical distance

For the fungal communities, Shannon indexes indicated that the samples from northwest China had higher biodiversity than those from the southeast (*p* < 0.001; Fig. 3a, Supplementary Figure S3e). The alpha diversity of fungal communities was mainly influenced by temperature (*p* = 0.009; Supplementary Figure S3f) but not altitude or humidity (*p* = 0.184 and 0.063; Supplementary Figure S3g, h). Beyond that, no significant difference between different insect superfamilies was observed according to the alpha diversity results (*p* = 0.134; Fig. 3b).

The PCoA plot showed a similar pattern to the alpha diversity results, in which fungal communities of insects collected from different regions were obviously differentiable, while no obvious pattern was shown across different superfamilies (Fig. 3c). According to the PERMANOVA test, the fungal communities in different insect superfamilies did not hold up significant difference (Supplementary Table S 5). This result was proved by both Mantel and Procrustes tests (*p* > 0.05; Fig. 3d, f). Besides, the PERMANOVA test also indicated that samples collected from the same or close sites might contain similar symbiotic fungal communities and show more differences from the distant ones (Supplementary Table S 6). The Procrustes test displayed a similar result that fungal community structures of true bugs were significantly correlated with the distance of living regions (*p* < 0.001; Fig. 3g). Maybe because solely considering the distance would distort the influence of important ecological factors like temperature, the geographic distance result displayed of Mantel test was different from that of Procrustes test.

### Evolutionary history of dominant symbiotic bacteria in true bugs

**Fig. 4:**
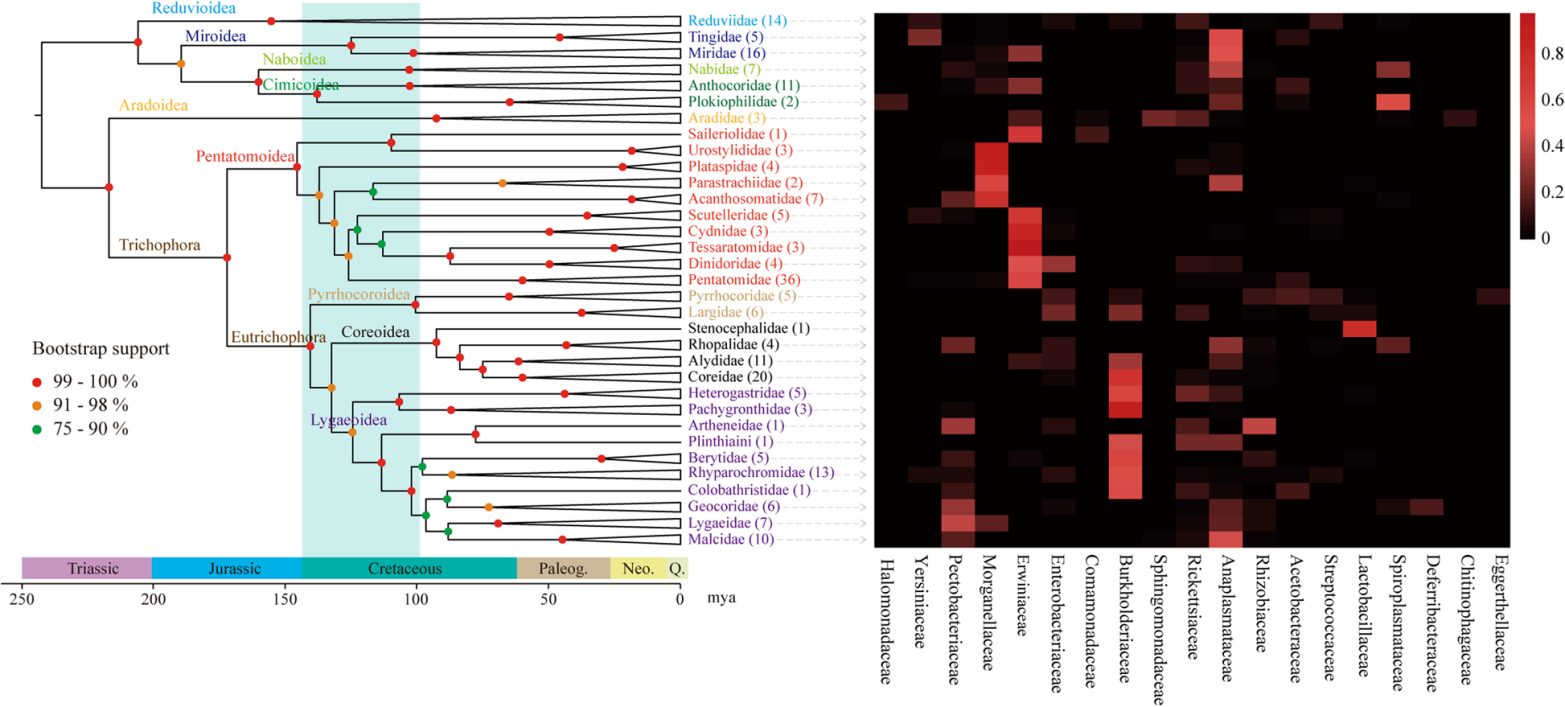
Dated phylogenetic tree of true bug families and the corresponding heatmap of their symbiotic bacterial communities at the family level. Colored circles indicate the bootstrap support values. Timescale in millions of years (bottom) are estimated by 10 fossil records. The number of species sampled within each family is indicated in parentheses. The light green block on the phylogenetic tree stands for the early Cretaceous which lasts from about 144.2 to 98.9 mya. The heatmap represents the most abundant 19 symbiotic bacteria families which are one-to-one matched with the phylogenetic tree

In order to elucidate the coevolution relationship between symbiotic bacteria and hosts, the phylogenetic relationships across Pentatomomorpha and Cimicomorpha were reconstructed using 18S rRNA, 28S rRNA, COI, and COII genes from 225 samples of 32 families. The phylogram and heatmap in Fig. 4 showed that closely related families generally contain similar bacteria families. In Miridae and its closely related families Tingidae and Nabidae, *Anaplasmataceae* could be found taking the largest proportion of the symbiotic bacterial communities. *Erwiniaceae* took the largest part of bacterial communities in the families Pentatomidae, Scutelleridae, Cydnidae, Tessaratomidae, and Dinidoridae, which formed a monophyletic group within Pentatomoidea. Besides, the stem families situated at the base of Pentatomoidea usually contained major bacteria belonging to *Morganellaceae*. The bacterial communities in the families of Pyrrhocoroidea, Coreoidea, and Lygaeoidea were generally dominated by *Burkholderiaceae* (Fig. 4).

**Fig. 5:**
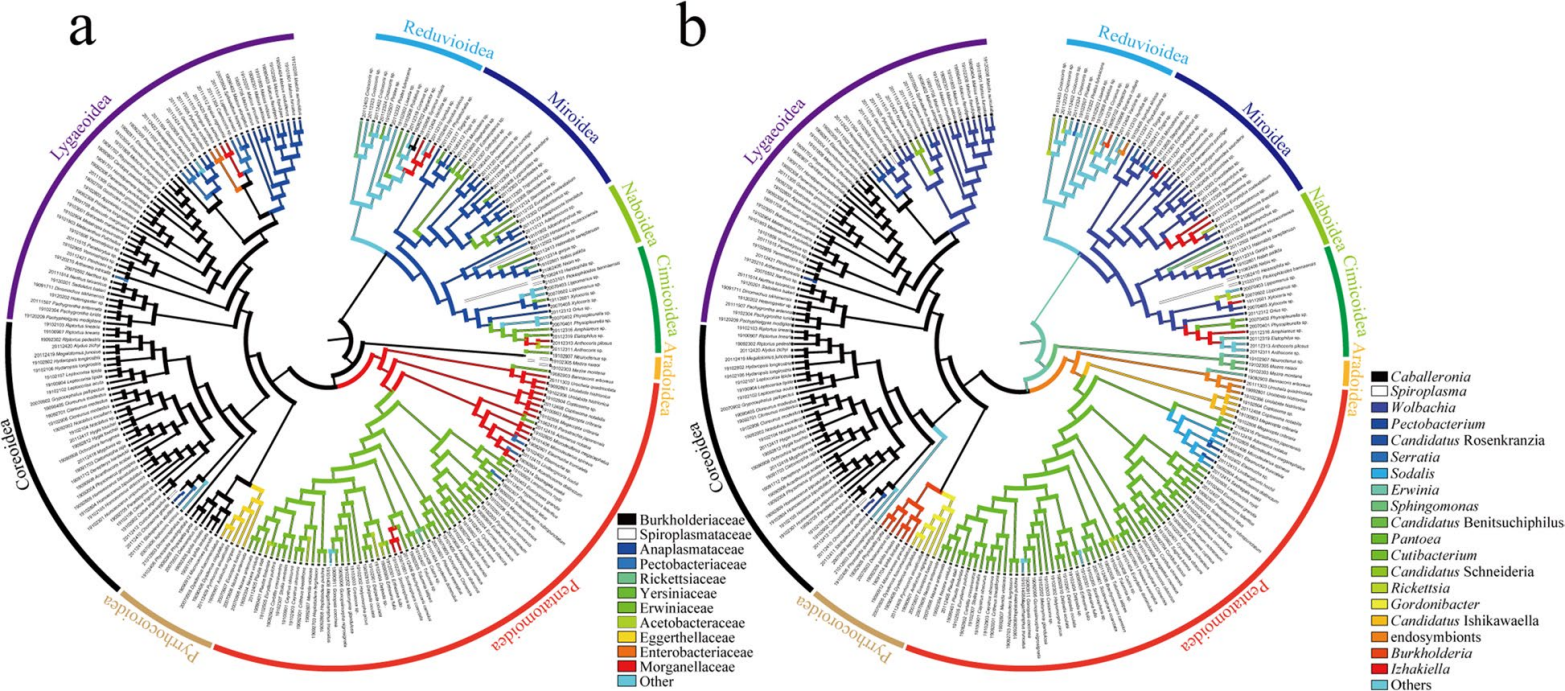
The ancestral state reconstructions indicating the replacements of dominant bacteria in the evolutionary history. The ancestral state reconstructions are carried out using Mesquite with the parsimony method. The dominant symbiotic bacteria families (**a**) or genera (**b**) are displayed as a character in this analysis. The phylogenetic relationships of researched true bugs are shown by the circle map. The color lines in the phylogenetic tree stand for the dominant symbiotic bacteria at the family or genus level of true bugs. The symbiotic bacteria families or genera that were only detected in one sample were classified as others

Based on the well-resolved and supported phylogram, the divergence time estimation calibrated by 10 fossil records was carried out (Supplementary Table S 7). In addition, based on the result that the symbiotic bacterial communities were mainly determined by insect hosts, we further reconstructed the ancestral states of dominant bacteria to trace back the symbiotic relationships between the bacteria and their hosts in the long evolutionary history (Fig. 5, Supplementary Figure S4). The dominant symbiotic bacterial genera and their basic information in different insect families can be found in Supplementary Table S8. According to the results, *Wolbachia* had been regarded as the dominant bacteria of the last common ancestor of Miroidea, Naboidea, and Cimicoidea, and accompanied the differentiation of Miroidea which occurred about 125.3 million years ago (mya) (Figs. 4 and 5b, Supplementary Figure S4b). The nearest common ancestor of Pentatomoidea was symbiotic with the genera of *Morganellaceae* and replaced by *Pantoea* before the divergence of the crown group including Pentatomidae, Scutelleridae, Cydnidae, Tessaratomidae, and Dinidoridae about 126.3 mya (Figs. 4 and 5a, Supplementary Figure S4a). The last common ancestor of Pyrrhocoroidea, Coreoidea, and Lygaeoidea was symbiotic with *Caballeronia*, while the dominant bacteria of Pyrrhocoridae and Largidae were replaced by *Paraburkholderia* and the genera of *Coriobacteriales* respectively after the diversification about 100.8 mya. In addition, *Caballeronia* had also been gradually replaced by other dominant symbiotic bacteria about 102.4 mya in the terminal families of Lygaeoidea (Figs. 4 and 5a, Supplementary Figure S4b).

## Discussion

In order to address the coevolution of insect hosts and their symbiotic bacteria, several studies have been carried out in diverse insect orders [11, 16, 23, 25, 37–39]. Among them, excellent works about how the hosts and their symbiotic bacteria interaction have been carried out in the plant-feeding true bugs [16, 23, 25, 37]. In this study, we sampled 209 species from 32 families, among which 10 families of phytophagous true bugs were studied for microbiome survey for the first time. Furthermore, we simultaneously address the lack of fungal research on plant-feeding true bugs which have been reported to play important roles as well in insects [40]. Therefore, full-length amplicons of both bacteria and fungi sequenced by the PacBio platform had been adopted to promote the knowledge of symbiotic microorganisms in the plant-feeding true bugs.

The major symbiotic bacteria revealed by this work were similar to those of the studied true bug families [16, 18, 23, 41], whereas the families and subfamilies that had never been involved in previous studies still displayed some fresh results. In Coreoidea, the family Rhopalidae showed a lack of dominant bacteria, in contrast to the other families of Coreoidea which were dominated by *Caballeronia* [24, 26]. Reduviidae was suggested to contain distinct major bacteria in each of the newly sampled five subfamilies. Together with the former studies which were restricted to Triatominae [42–44], this family may lack stable symbiotic relationships with specific bacteria groups. Besides, the species of Plokiophilidae, usually living with spiders on cobwebs [45], had distinct bacterial communities compared with those of Anthocoridae which was similar to the other closely related families in Cimicoidea. These first researched groups have helped to expand our knowledge of symbiotic microorganisms in true bugs.

It is noteworthy that both hosts and habitats significantly influence the symbiotic bacterial communities of phytophagous true bugs but with different roles, in which the hosts mainly affect the dominant bacteria, while the habitats generally influence the subordinate bacteria. For this reason, the beta diversity and its related statistical analyses showed the symbiotic bacterial communities were more relevant to the hosts than to the ecological factors in this study. For a typical example, both samples of *Megacopta cribraria* (Pentatomoidea: Plataspidae) from Yunnan and Guangdong contained *Candidatus* Ishikawaella as the dominant symbiotic bacteria, whereas different subordinate bacteria were involved in their bacterial communities. Consistently, accumulated studies of Pentatomidae have indicated that *Pantoea* is the dominant symbiotic bacteria no matter sampled in Europe [36], the Middle East [46], East Asia [21] or North America [47]. Mechanisms like vertical transmission and symbiont-mediated morphogenesis may be the reasons causing the specificity of dominant bacteria in plant-feeding true bugs. According to the former studies, the dominant bacteria in the superfamilies Miroidea and Pentatomoidea have been regarded as vertical transmission which makes these dominant bacteria stably transmitted to the offspring [19, 29, 48]. Besides, although some dominant bacteria like *Burkholderiaceae* in Pyrrhocoroidea, Coreoidea, and Lygaeoidea are not strictly vertically transmitted [49, 50], the symbiont-mediated morphogenesis still exists to maintain host-symbiont specificity in symbiosis [35].

Despite the existing mechanisms to maintain the host-symbiont specificity, dramatic replacements of the dominant symbiotic bacteria in the plant-feeding true bugs were still observed in the evolutionary history. In Pentatomoidea, the dominant bacteria in the last common ancestors of the crown families were replaced from the genera of *Morganellaceae* to the genus *Pantoea* of *Erwiniaceae*, which were inherited by the descendent families including Pentatomidae and other four families. In the nearest common ancestor of the superfamily Pyrrhocoroidea, *Caballeronia* was lost and the *Paraburkholderia* or the genera of *Coriobacteriales* were respectively acquired as dominant symbionts in Largidae and Pyrrhocoridae accompanying their differentiation. Similarly, *Caballeronia* was also lost and replaced by other dominant bacteria in three-terminal families of Lygaeoidea. All of these replacements were accompanied by the family-level differentiations of phytophagous true bugs and formed new stable symbiotic relationships. We can also find that not only the dominant symbionts had been replaced, but the localization and transmission modality have been changed as well (see Supplementary Table S8). On the whole, the localizations of dominant symbionts have been changed from the bacteriocyte or bacteriome of Miroidea to midgut crypts of Pentatomoidea, Pyrrhocoroidea, Coreoidea, and Lygaeoidea. The transmission modalities have been changed from strict vertical transmission to non-strict vertical transmission then to acquiring from the environment by symbiont-mediated morphogenesis. These results suggest that the dependence levels of phytophagous true bugs on dominant symbionts are largely decreasing from base to terminal in the phylogenetic tree of Pentatomomorpha, but with a few exceptions in Lygaeoidea.

According to the results of divergence time estimation and the ancestral state reconstruction, almost all of the mentioned replacements at the family level of plant-feeding true bugs intensively happened in the early Cretaceous (about 144.2–98.9 mya) [51], which is also the timespan for the explosion of Angiosperm orders [52]. Interestingly, insect diversification has also been shown to correlate with the radiation of flowering plants [53, 54]. Previous studies have revealed that plants have raised various challenges to phytophagous insects, which at least include complex plant polymers digestion, low and imbalanced amino acid profiles, and toxic chemicals protection [55–57]. Besides the adaptations based on insects themselves, the symbionts also make crucial contributions to break down plant polymers, supplement limiting nutrients, or detoxify plant defense compounds, thereby directly impacting the insect’s ability to exploit certain host plants as nutritional resources [11, 58–60]. Interestingly, the changing of host plants would often accompany the changing of symbionts [61]. Hence, the newly acquired dominant bacteria may act as powerful allies of plant-feeding true bugs to occupy formerly unsuitable ecological niches, thus explaining the intensive replacements of dominant bacteria during the adaptive radiation of the phytophagous true bugs in the early Cretaceous.

Besides, as shown in the ancestral state reconstruction results, the ancestors of Pyrrhocoroidea, Coreoidea, and Lygaeoidea share the same dominant symbiotic bacteria family which is different from that of Pentatomoidea, which indicates a close relationship among Pyrrhocoroidea, Coreoidea, and Lygaeoidea. Furthermore, we can also find that the ancestors of Coreoidea and Lygaeoidea share the same dominant symbiotic bacteria genus which is different from that of Pyrrhocoroidea. This result may imply that Coreoidea is closer to Lygaeoidea than to Pyrrhocoroidea.

For the revealed symbiotic fungal communities, the phyla *Ascomycota* and *Basidiomycota*, especially the genus *Cladosporium*, took the largest proportion of the plant-feeding true bugs which were similar to the other Hemiptera insects [62, 63]. These fungi would often provide nutrition or antimicrobial defense functions which are important to insect hosts [40]. According to the statistical results, symbiotic fungal communities of true bugs were mainly influenced by the temperature. Moreover, evidence in this study also indicated that fungal communities would not be significantly influenced by hosts. Compared with the case of bacteria, the fungal community of *Megacopta cribraria* (Pentatomoidea: Plataspidae) in Yunnan was different from that in Guangdong, while it was more similar to that of *Cletus trigonus* (Coreoidea: Coreidae), a species collected at the same sample site but situated far away from *Megacopta cribraria* in the phylogenetic tree. The result displayed in this study that the same or closely related species contained different fungal communities, is obviously different from the pattern of other vertical transmission symbiotic fungi [64]. In addition, the fungal genera taking the highest proportion, including *Cladosporium*, *Nigrospora*, *Alternaria*, and *Eremothecium*, have been previously reported as existing in insect bodies but acquired from the environment [65–68]. Therefore, based on this evidence, the transmission mode of symbiotic fungi in true bugs is regarded as mainly horizontal transmission.

## Conclusion

In summary, our study surveyed almost all higher categories of plant-feeding true bugs and their closely related groups for the first time, which summarized the symbiotic patterns of both bacteria and fungi. The symbiotic bacterial communities are influenced by both hosts and environment, in which the dominant bacteria are mainly determined by the former, and the subordinate ones are generally influenced by the latter. The dramatic replacements of dominant bacteria in the plant-feeding true bugs may help to drive the adaptive radiation of plant-feeding true bugs in the early Cretaceous. In contrast to bacteria, symbiotic fungi in plant-feeding true bugs are probably acquired from the environment through horizontal transmission mode which makes the fungal communities mainly influenced by the environment but not hosts. These conclusions help to provide a general evolutionary and ecological pattern of the symbiotic relationship between plant-feeding insects and their symbionts.

## Methods

### Sampling and DNA extracting

In order to characterize the microbial communities of the plant-feeding true bugs and infer their phylogeny relationship, adult specimens of 209 species belonging to 32 families were collected from their respective habitats all over China from 2019 to 2021 (Supplementary Table S1 and Supplementary Figure S1). The ecological factors including temperature, humidity, and altitude of samples were identified by China’s eco-geographical region map [69]. Besides, the Heihe-Tengchong Line, which is a natural population and geographical boundary of China, was used to classify the samples geographically [70]. Insects collected in the field were preserved in tubs filled with ethanol until further analysis, and one individual per sample was kept as a voucher specimen. The method to extract the DNA from insect symbiotic microorganisms was carried out according to the former study [71]. The DNA of insect leg homogenate was extracted by the TIANamp Micro DNA Kit (TIANGEM, Beijing, China) following the manufacturer’s instructions for the host phylogenetic analyses.

### Symbiotic microbial community analysis

The symbiotic microbial communities were surveyed using full-length 16S rRNA gene and ITS amplicons sequenced by PacBio Sequel II platform in the Biomarker Technologies [72, 73]. All used primers and the annealing temperatures were listed in Supplementary Table S2. The obtained circular consensus sequencing (CCS) reads from the PacBio platform were controlled quality and removed chimeras by the DADA2 package (v.1.18) [74] in R (v.4.0.4) to get the ASV tables and representative sequences. The files obtained from DADA2 were imported into QIIME2 (v.2021.4) [75] for further analysis. The ASVs were filtered by q2-feature-table plugin when contenting less than 50 sequences for bacterial sequences or 30 sequences for fungal sequences to avoid possible contamination or other false-positive results. Representative sequences were assigned taxonomies by the q2-feature-classifier plugin using a pre-fitted sklearn-based taxonomy classifier method [76]. The SILVA database (v.138.1) [77] and the UNITE database (v.8.2) [78] were used to annotate the ASV tables for bacteria and fungi respectively. The q2-diversity plugin was used to calculate the alpha and beta diversity indexes. The package ggplot2 was used to create PCoA plots using the weighted Unifrac distance matrix [79]. The Kruskal–Wallis test and PERMANOVA test were used to statistically compare the differences between different sample groups [80, 81]. The Mantel and Procrustes tests were performed using the vegan package, which can be used to verify the correlations between the symbiotic microbial communities and the hosts or environment by the weighted Unifrac distances of symbiotic microbial communities and the genetic distance of hosts or the geographical distance of sample sites [82–84].

### Host phylogeny reconstruction

Phylogenetic analyses were conducted using the nearly complete nuclear genes (18S nrDNA and 28S nrDNA) and mitochondrial genes (COI and COII). The nucleotide sequences of protein-coding genes (COI and COII) were translated into amino-acid sequences. All nucleotide and amino-acid sequences were preliminarily aligned using Mafft (v.7.475) [85]. Then, the aligned nrDNAs were checked and manually corrected according to the secondary structure models of 18S rRNA and 28S rRNA respectively [86]. All these alignments were concatenated using the SequenceMatrix (v.1.78) [87]. The software ModelFinder [88], which is embedded within IQ-TREE (v.1.6.12) [89], was used to infer the best substitution models for the nrDNAs and the mitochondrial genes. Phylogenetic analysis was conducted with RAxML (v.8.2.8) using the maximum likelihood (ML) method [90]. For ML analysis, the substitution model GTR + G + I was used for the nucleotides partition and the substitution model mtZOA + G + I was used for the amino acids partition. The best ML tree was calculated with 1000 replicates. Bootstrap values were calculated by the software BOOSTER (v.0.1.2) [91] with default settings.

### Divergence time estimation of hosts

The divergence time of insect hosts was estimated based on the hybrid matrix using the program BEAST (v.2.5.2) [92]. The nrDNAs and the mitochondrial genes were considered as one matrix and the amino acids of COI and COII were considered as the second matrix. Both of them were loaded into BEAUti simultaneously. The substitution models used in the divergence time estimations were consistent with those used in the phylogenetic reconstruction. A relaxed molecular clock model with log-normal distribution, which took into account the variation of the substitution rate among branches, was adopted [93]. The birth–death skyline contemporary was selected as a tree prior to the prior sets [94]. Ten fossil species from various lineages were used to calibrate the internal nodes with soft boundaries (Supplementary Table S7).

The BEAST analysis was run for a total of 100,000,000 generations and was sampled every 100 generations. Tracer (v.1.7) [95] was used to examine the posterior distribution of all parameters and their associated statistics, such as the effective sample size (ESS) and the 95% high posterior density (HPD) intervals. All of the ESS values were above the recommended threshold of 200, indicating that the parameter space had been sufficiently sampled.

### Ancestral state reconstruction

In order to explore the symbiotic relationship between dominant bacteria and phytophagous true bugs in evolutionary history, the ancestral state of the dominant bacteria in the insect hosts was reconstructed using Mesquite (v.3.70 http://www.mesquiteproject.org). The bacteria were defined as the dominant if they were previously reported as the main symbionts or not reported but occupied the highest proportion of the samples in this study. The dominant families or genera detecting only in one sample were classified as others. Both the parsimony ancestral character and the Markov k-state 1 parameter model of likelihood ancestral character were used to do the reconstruction work. The ancestral state was considered reliable if this state took the proportional likelihood percentage higher than 75% in the node.

### Supplementary Information

**Additional file 1: Fig. S1.** The sample sites distribution around China. **Fig. S2. **Composition of symbiotic bacterial and fungal communities in plant-feeding true bugs at the phylum level. **Fig. S3. **The differences of alpha diversity results for different ecological factors. **Fig. S4. **The ancestral state reconstructions indicating the replacements of dominant bacteria in the evolutionary history by likelihood method. **Table S1. **The phylogeny and environmental factor information of studied insect samples. **Table S2. **The primers used in this study. **Table S3. **The results of PERMANOVA test of the symbiotic bacterial communities in different insect superfamilies. **Table S4. **The results of PERMANOVA test of the symbiotic bacterial communities in different insect superfamilies. **Table S5. **The results of PERMANOVA test of the symbiotic fungal communities in different insect superfamilies. **Table S6. **The results of PERMANOVA test of the symbiotic fungal communities in different sample sites. **Table S7. **The fossil calibrations used in the divergence time estimation. **Table S8. **The insect host families and their dominant symbionts.

## Data Availability

The amplicon data are available in the CNCB-NGDC (The National Genomics Data Center (NGDC), part of the China National Center for Bioinformation (CNCB), https://ngdc.cncb.ac.cn/) under accession numbers CRA005829 and CRA005851. The genome sequences of insect hosts are achieved available from the GenBank under the accession numbers OM276296–OM276504, OM276508–OM276725, OM323091–OM323308, and OM321106–OM321305.
