# Supplementary material for: Different roles of host and habitat in determining the microbial communities of plant-feeding true bugs
Source: Microbiome. 2023 Nov 7;11:244. doi: 10.1186/s40168-023-01702-y (PMC10629178; doi:10.1186/s40168-023-01702-y)
Supplement: Supplementary file 2 — Additional file 1: Fig. S1. The sample sites distribution around China. Fig. S2. Composition of symbiotic bacterial and fungal communities in plant-feeding true bugs at the phylum level. Fig. S3. The differences of alpha diversity results for different ecological factors. Fig. S4. The ancestral state reconstructions indicating the replacements of dominant bacteria in the evolutionary history by likelihood method. Table S1. The phylogeny and environmental factor information of studied insect samples. Table S2. The primers used in this study. Table S3. The results of PERMANOVA test of the symbiotic bacterial communities in different insect superfamilies. Table S4. The results of PERMANOVA test of the symbiotic bacterial communities in different insect superfamilies. Table S5. The results of PERMANOVA test of the symbiotic fungal communities in different insect superfamilies. Table S6. The results of PERMANOVA test of the symbiotic fungal communities in different sample sites. Table S7. The fossil calibrations used in the divergence time estimation. Table S8. The insect host families and their dominant symbionts. [file 40168_2023_1702_MOESM1_ESM.docx]

**Supplementary Information for**

**Different roles of** **host and habitat in** **determining the** **microbial communities of plant-feeding true bugs**

Zi-Wen Yang^1^, Jiu-Yang Luo^1^, Yu Men^2^, Zhi-Hui Liu^1^, Zi-Kai Zheng^1^, Yan-Hui Wang^1^ and Qiang Xie^1*^

1. School of Life Sciences, State Key Laboratory of Biocontrol, Sun Yat-sen University, Guangzhou 510275, Guangdong, China
2. School of Life Sciences, Zhaoqing University, Zhaoqing 526061, China

Correspondence:

Qiang Xie, Sun Yat-sen University, Guangzhou, 510275, China

Email: [xieq8@mail.sysu.edu.cn](mailto:xieq8@mail.sysu.edu.cn)


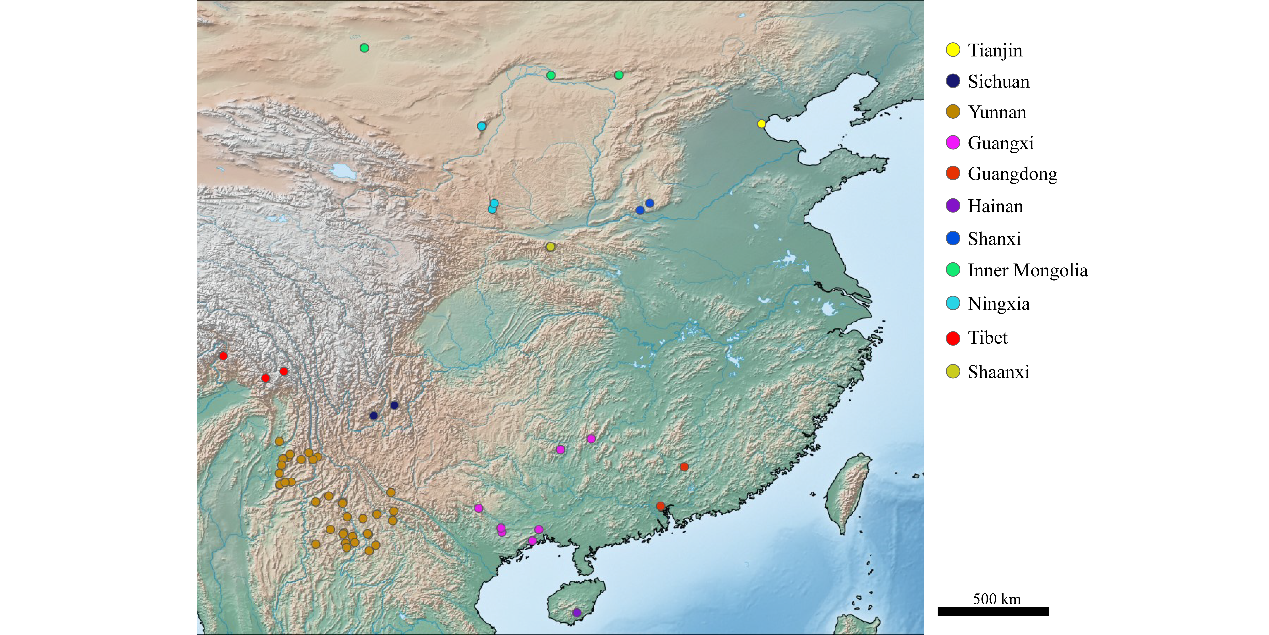


**Supplementary Fig. S1 The sample sites distribution around China.** The samples are grouped according to their located provinces of China and marked by different colors.


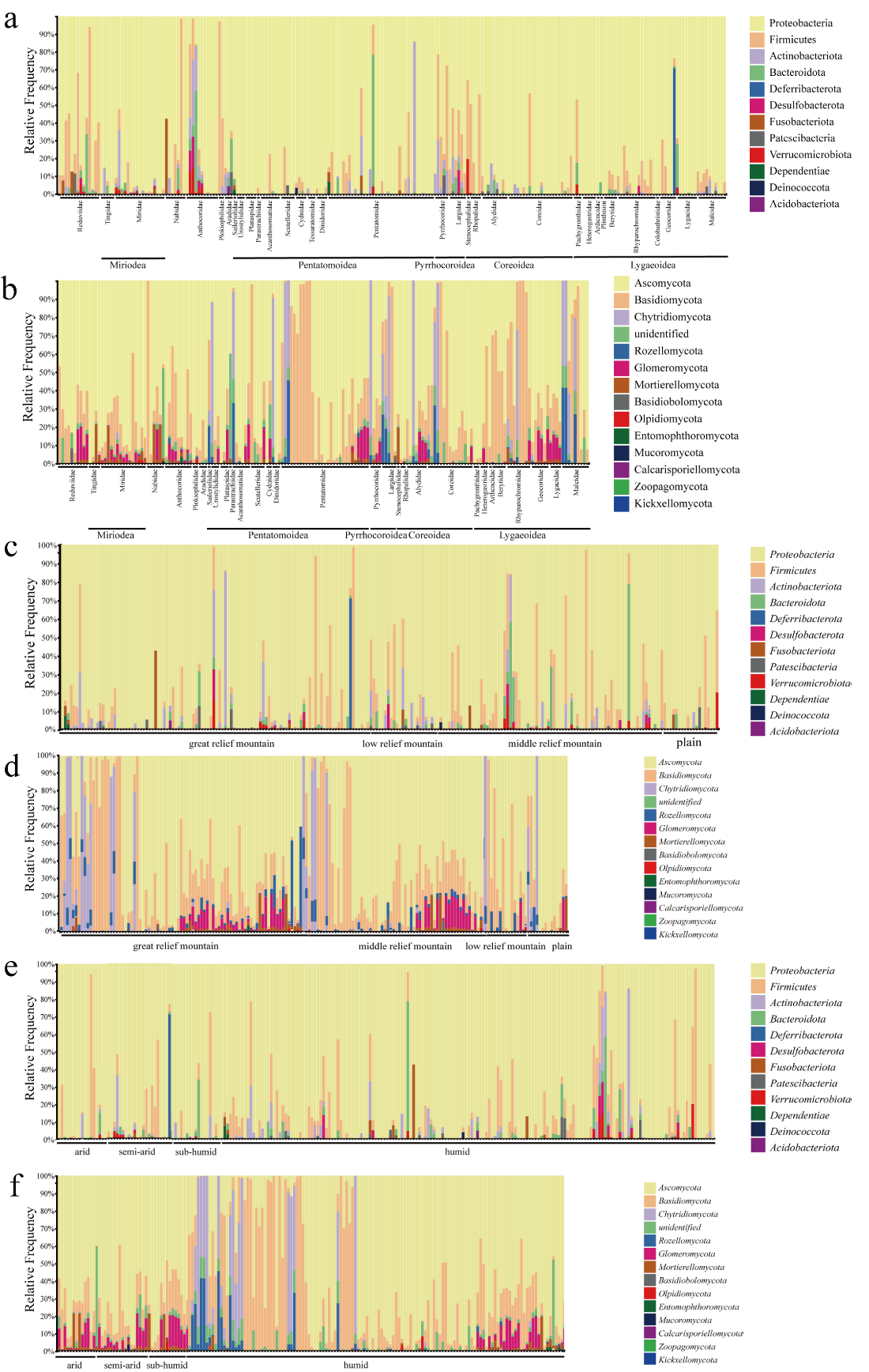


**Supplementary Fig. S2 Composition of symbiotic bacterial and fungal communities in plant-feeding true bugs at the phylum level.** The relative abundance plots of bacteria (a, c, and e, n=225) and fungi (b, d, and f, n= 174) are displayed. Totally 12 phyla in bacterial communities and 14 phyla in fungal communities are shown, which represent the most abundant phyla. The samples are grouped according to the hosts and listed according to their phylogenetic relationship. Besides, the five phytophagous true bug superfamilies, altitude and humid are shown at the bottom of plots.


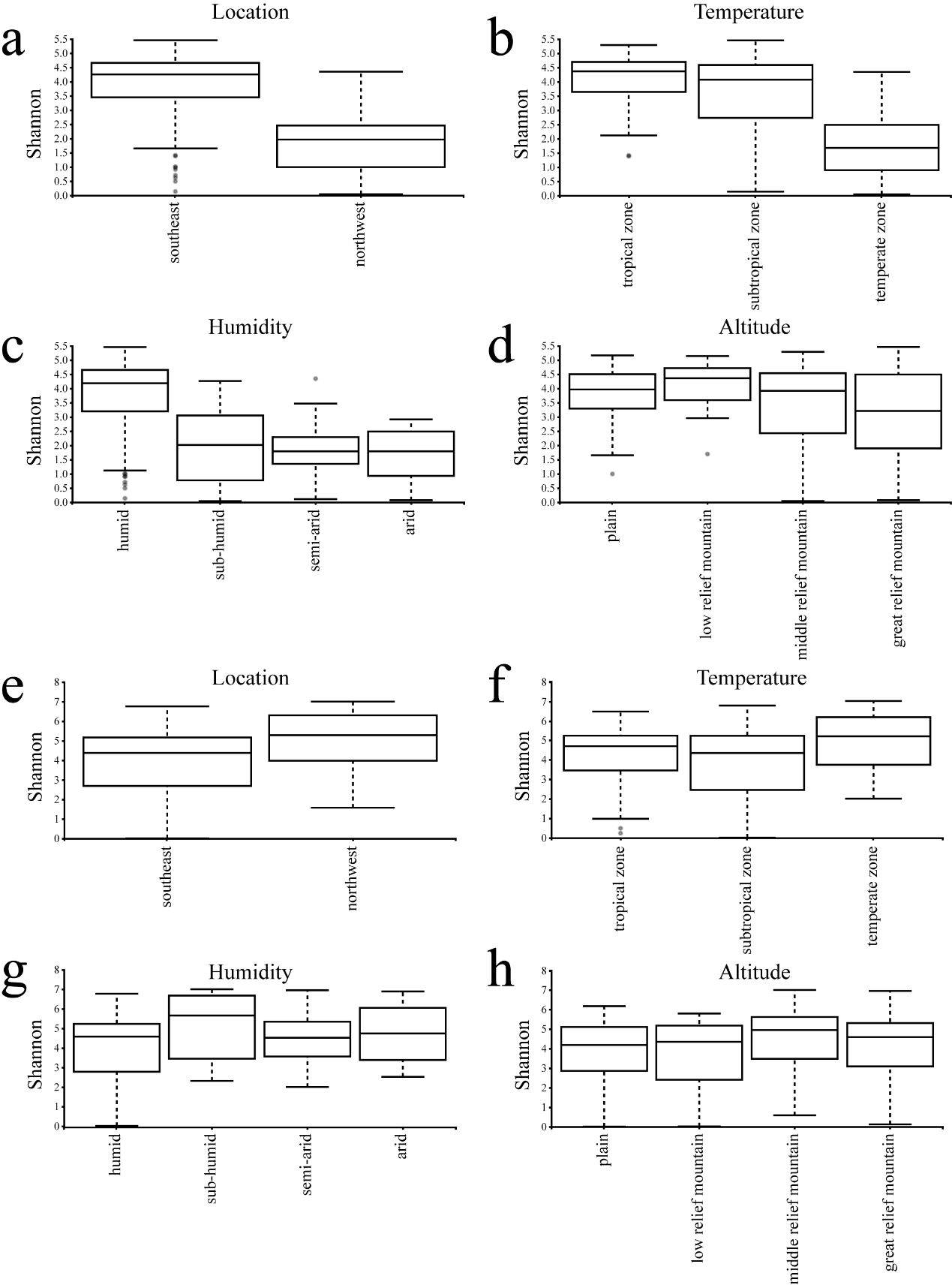


**Supplementary Fig. S3** **The differences of alpha diversity results for different ecological factors.** a-d, The alpha diversity results of bacterial communities. e-h, The alpha diversity results of fungal communities. The sample locations are divided into southeast and northwest of China according to the Heihe-Tengchong Line. The ecological factors of samples were identified by the China’s eco-geographical region map.


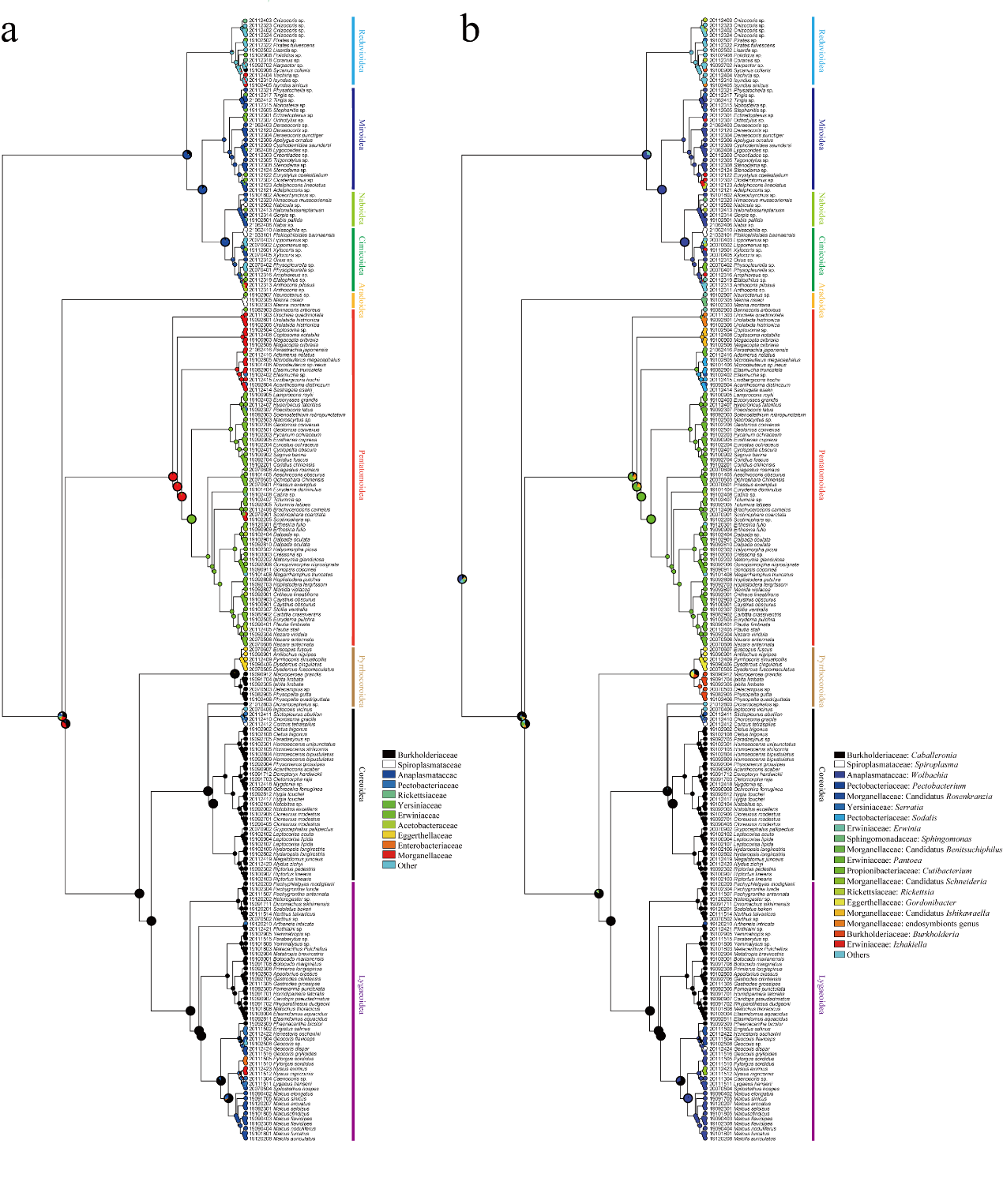


**Supplementary Fig. S4** **The ancestral state reconstructions indicating the replacements of dominant bacteria in the evolutionary history by likelihood method**. The ancestral state reconstructions are carried out using Mesquite with likelihood method. The dominant symbiotic bacteria families (a) or genera (b) are displayed as a character in this analysis. The proportion of different colors in the circles stand for the reliability of the dominant symbiotic families or genera in each node. The ancestral states are considered reliable if taking the proportional higher than 75% in these nodes. The symbiotic bacteria families or genera which were only detected in one sample were classified as others.

**Supplementary Table S1. The phylogeny and environmental factor information of studied insect samples.**

| Sample  ID | Infraorder | Superfamily | Family | species | collection data | Temperature | humidity | location | longitude and latitude | Altitude |
| --- | --- | --- | --- | --- | --- | --- | --- | --- | --- | --- |
| 20112323 | Cimicomorpha | Reduvioidea | Reduviidae | *Cnizocoris* sp. | 2020.8.25 | temperate | sub-humid | Shanxi | 35°33'47''N 112°50'15''E | 1001m |
| 20112324 | Cimicomorpha | Reduvioidea | Reduviidae | *Cnizocoris* sp. | 2020.7.31 | temperate | arid | Ningxia | 38°44'23''N 105°56'25''E | 1730m |
| 20112402 | Cimicomorpha | Reduvioidea | Reduviidae | *Cnizocoris* sp. | 2020.7.27 | temperate | semi-arid | Ningxia | 35°19'2''N 106°21'3''E | 1762m |
| 20112403 | Cimicomorpha | Reduvioidea | Reduviidae | *Cnizocoris* sp. | 2020.8.11 | temperate | semi-arid | Inner Mongolia | 40°50'4''N 101°32'45''E | 1080m |
| 20112318 | Cimicomorpha | Reduvioidea | Reduviidae | *Coranus* sp. | 2020.8.17 | subtropical | humid | Shaanxi | 33°46'8''N 108°48'23''E | 1334m |
| 19092702 | Cimicomorpha | Reduvioidea | Reduviidae | *Harpactor* sp. | 2019.8.8 | tropical | humid | Yunnan | 23°30'43''N 98°5'7''E | 440m |
| 19102405 | Cimicomorpha | Reduvioidea | Reduviidae | *Isyndus sinicus* | 2020.7.30 | tropical | humid | Yunnan | 22°8'9''N 99°40'55''E | 940m |
| 20112310 | Cimicomorpha | Reduvioidea | Reduviidae | *Isyndus* sp. | 2020.8.18 | subtropical | humid | Tibet | 29°16'54''N 95°15'22''E | 672m |
| 19102502 | Cimicomorpha | Reduvioidea | Reduviidae | *Lisarda* sp. | 2019.7.20 | tropical | humid | Yunnan | 21°56'6''N 101°15'6''E | 569m |
| 20112322 | Cimicomorpha | Reduvioidea | Reduviidae | *Pirates fulvescens* | 2020.8.21 | temperate | sub-humid | Shanxi | 35°16'25''N 112°26'27''E | 658m |
| 19102507 | Cimicomorpha | Reduvioidea | Reduviidae | *Pirates* sp. | 2019.8.5 | tropical | humid | Yunnan | 23°16'26''N 99°4'26''E | 566m |
| 19102908 | Cimicomorpha | Reduvioidea | Reduviidae | *Polididus* sp. | 2019.7.16 | subtropical | humid | Guangxi | 22°8'0''N 108°16'10''E | 2m |
| 19100906 | Cimicomorpha | Reduvioidea | Reduviidae | *Sycanus collaris* | 2019.9.13 | subtropical | humid | Guangxi | 22°11'57''N 106°42'29''E | 100m |
| 20112404 | Cimicomorpha | Reduvioidea | Reduviidae | *Vachiria* sp. | 2020.8.8 | temperate | arid | Inner Mongolia | 40°49'43''N 108°46'9''E | 996m |
| 20112315 | Cimicomorpha | Miroidea | Tingidae | *monosteira* sp. | 2020.8.4 | temperate | arid | Inner Mongolia | 41°57'8''N 101°5'10''E | 888m |
| 20112321 | Cimicomorpha | Miroidea | Tingidae | *Physatocheila* sp. | 2020.7.31 | temperate | arid | Ningxia | 38°44'23''N 105°56'25''E | 1730m |
| 19112605 | Cimicomorpha | Miroidea | Tingidae | *Stephanitis* sp. | 2019.7.28 | tropical | humid | Yunnan | 21°50'14''N 100°35'11''E | 1878m |
| 20112317 | Cimicomorpha | Miroidea | Tingidae | *Tingis* sp. | 2020.8.11 | temperate | semi-arid | Inner Mongolia | 40°50'4''N 101°32'45''E | 1080m |
| 21062412 | Cimicomorpha | Miroidea | Tingidae | *Tingis* sp. | 2021.5.6 | subtropical | humid | Yunnan | 23°39′35″N 102°10′37″E | 1897m |
| 20112123 | Cimicomorpha | Miroidea | Miridae | *Adelphocoris lineolatus* | 2020.8.11 | temperate | semi-arid | Inner Mongolia | 40°50'4''N 111°32'45''E | 1089m |
| 20112121 | Cimicomorpha | Miroidea | Miridae | *Adelphocoris* sp. | 2020.7.28 | temperate | semi-arid | Ningxia | 35°33'32''N 106°25'50''E | 1762m |
| 20112306 | Cimicomorpha | Miroidea | Miridae | *Apolygus ornatus* | 2020.8.31 | temperate | arid | Ningxia | 38°44'23''N 105°56'25''E | 1730m |
| 20112302 | Cimicomorpha | Miroidea | Miridae | *Closterotomus* sp. | 2020.8.15 | subtropical | humid | Shaanxi | 33°46'8''N 108°48'24''E | 1334m |
| 20112303 | Cimicomorpha | Miroidea | Miridae | *Creontiades* sp. | 2020.8.27 | temperate | semi-arid | Ningxia | 35°19'2''N 106°21'3''E | 2191m |
| 20112309 | Cimicomorpha | Miroidea | Miridae | *Cyphodemidea saundersi* | 2020.7.27 | temperate | semi-arid | Ningxia | 35°19'2''N 106°21'3''E | 2191m |
| 21062404 | Cimicomorpha | Miroidea | Miridae | *Cyrtorhinus lividipennis* | 2021.5.18 | tropical | humid | Yunnan | 23°39′35″N 102°10′37″E | 744m |
| 20112304 | Cimicomorpha | Miroidea | Miridae | *Deraeocoris punctulatus* | 2020.8.11 | temperate | semi-arid | Inner Mongolia | 40°50'4''N 111°32'45''E | 1089m |
| 20112120 | Cimicomorpha | Miroidea | Miridae | *Deraeocoris* sp. | 2020.7.28 | temperate | semi-arid | Ningxia | 35°33'32''N 106°25'50''E | 1762m |
| 21062403 | Cimicomorpha | Miroidea | Miridae | *Deraeocoris* sp. | 2021.5.3 | subtropical | humid | Sichuan | 26°49′1″N 101°28′8″E | 1243m |
| 20112301 | Cimicomorpha | Miroidea | Miridae | *Ectmetopterus* sp. | 2020.8.21 | temperate | sub-humid | Shanxi | 35°16'25''N 112°26'27''E | 658m |
| 20112122 | Cimicomorpha | Miroidea | Miridae | *Eurystylus coelestialium* | 2020.8.22 | temperate | sub-humid | Shanxi | 35°14'34''N 112°28'17''E | 564m |
| 21062408 | Cimicomorpha | Miroidea | Miridae | *Lygocorides* sp. | 2021.4.24 | subtropical | humid | Sichuan | 27°15′48″N 102°19′7″E | 1278m |
| 20112307 | Cimicomorpha | Miroidea | Miridae | *Orthotylus* sp. | 2020.8.8 | temperate | arid | Inner Mongolia | 40°49'43''N 108°46'9''E | 996m |
| 20112124 | Cimicomorpha | Miroidea | Miridae | *Stenodema* sp. | 2020.8.19 | subtropical | humid | Tibet | 28°39'5''N 97°46'27''E | 3945m |
| 20112308 | Cimicomorpha | Miroidea | Miridae | *Stenodema* sp. | 2020.7.28 | temperate | semi-arid | Ningxia | 35°33'32''N 106°25'50''E | 1762m |
| 20112305 | Cimicomorpha | Miroidea | Miridae | *Trigonotylus* sp. | 2020.8.12 | temperate | semi-arid | Inner Mongolia | 40°50'50''N 111°34'24''E | 1117m |
| 19101802 | Cimicomorpha | Naboidea | Nabidae | *Alloeorhynchus* sp. | 2019.7.24 | subtropical | humid | Yunnan | 21°34'36''N 100°18'27''E | 1073m |
| 20112314 | Cimicomorpha | Naboidea | Nabidae | *Gorpis* sp. | 2020.8.3 | temperate | arid | Inner Mongolia | 41°57'8''N 101°5'10''E | 888m |
| 20112413 | Cimicomorpha | Naboidea | Nabidae | *Halonabis sareptanus* | 2020.8.6 | temperate | arid | Inner Mongolia | 41°57'53''N 101°4'31''E | 929m |
| 20112320 | Cimicomorpha | Naboidea | Nabidae | *Himacerus mussooriensis* | 2020.7.28 | temperate | semi-arid | Ningxia | 35°33'32''N 106°25'50''E | 1762m |

continued

| 20112502 | Cimicomorpha | Naboidea | Nabidae | *Nabicula* sp. | 2020.7.28 | temperate | semi-arid | Ningxia | 35°33'32''N 106°25'50''E | 1762m |
| --- | --- | --- | --- | --- | --- | --- | --- | --- | --- | --- |
| 19102801 | Cimicomorpha | Naboidea | Nabidae | *Nabis pallida* | 2019.9.29 | subtropical | sub-humid | Tianjin | 38°49'39''N 117°26'53''E | 0m |
| 21062406 | Cimicomorpha | Naboidea | Nabidae | *Nabis* sp. | 2021.4.24 | subtropical | humid | Sichuan | 27°15′48″N 102°19′7″E | 1278m |
| 20112316 | Cimicomorpha | Cimicoidea | Anthocoridae | *Amphiareus* sp. | 2020.8.22 | temperate | sub-humid | Shanxi | 35°14'34''N 112°28'17''E | 564m |
| 20112313 | Cimicomorpha | Cimicoidea | Anthocoridae | *Anthocoris pilosus* | 2020.7.30 | temperate | arid | Ningxia | 38°44'10''N 105°54'41''E | 2283m |
| 20112311 | Cimicomorpha | Cimicoidea | Anthocoridae | *Anthocoris* sp. | 2020.8.27 | temperate | semi-arid | Ningxia | 35°19'2''N 106°21'3''E | 2191m |
| 20112319 | Cimicomorpha | Cimicoidea | Anthocoridae | *Elatophilus* sp. | 2020.8.1 | temperate | arid | Ningxia | 38°44'56''N 105°54'59''E | 1937m |
| 20070403 | Cimicomorpha | Cimicoidea | Anthocoridae | *Lippomanus* sp. | 2020.5.25 | tropical | humid | Hainan | 18°42'35''N 109°50'28''E | 570m |
| 20070602 | Cimicomorpha | Cimicoidea | Anthocoridae | *Lippomanus* sp. | 2020.5.20 | subtropical | humid | Guangxi | 23°0'42''N 105°47'28''E | 225m |
| 20112312 | Cimicomorpha | Cimicoidea | Anthocoridae | *Orius* sp. | 2020.8.12 | temperate | semi-arid | Inner Mongolia | 40°50'50''N 111°34'24''E | 1113m |
| 20070401 | Cimicomorpha | Cimicoidea | Anthocoridae | *Physopleurella* sp. | 2020.5.1 | tropical | humid | Yunnan | 22°45'43''N 101°35'47''E | 710m |
| 20070402 | Cimicomorpha | Cimicoidea | Anthocoridae | *Physopleurella* sp. | 2020.5.4 | tropical | humid | Yunnan | 22°29'59''N 102°15'6''E | 1140m |
| 19112601 | Cimicomorpha | Cimicoidea | Anthocoridae | *Xylocoris* sp. | 2019.9.7 | subtropical | humid | Yunnan | 23°13'4''N 100°11'18''E | 1650m |
| 20070405 | Cimicomorpha | Cimicoidea | Anthocoridae | *Xylocoris* sp. | 2020.5.5 | tropical | humid | Yunnan | 22°35'0''N 102°17'14''E | 320m |
| 21062410 | Cimicomorpha | Cimicoidea | Plokiophilidae | *Heissophila* sp. | 2021.5.16 | tropical | humid | Yunnan | 21°56'6''N 101°15'6''E | 545m |
| 21033101 | Cimicomorpha | Cimicoidea | Plokiophilidae | *Plokiophiloides bannaensis* | 2021.5.13 | tropical | humid | Yunnan | 21°56'6''N 101°15'6''E | 1943m |
| 19102907 | Pentatomomorpha | Aradoidea | Aradidae | *Neuroctenus* sp. | 2019.8.7 | tropical | humid | Yunnan | 25°28'23''N 98°57'47''E | 1320m |
| 19102303 | Pentatomomorpha | Aradoidea | Aradidae | *Mezira montana* | 2019.7.26 | tropical | humid | Yunnan | 21°37'32''N 100°30'5''E | 1188m |
| 19102305 | Pentatomomorpha | Aradoidea | Aradidae | *Mezira hsiaoi* | 2019.7.26 | tropical | humid | Yunnan | 21°37'32''N 100°30'5''E | 1188m |
| 19082903 | Pentatomomorpha | Pentatomoidea | Saileriolidae | *Bannacoris arboreus* | 2019.8.16 | subtropical | humid | Yunnan | 25°1'4''N 98°28'43''E | 1896m |
| 19092801 | Pentatomomorpha | Pentatomoidea | Urostylididae | *Urolabida histrionica* | 2019.7.24 | tropical | humid | Yunnan | 21°34'24''N 100°21'9''E | 1222m |
| 19102306 | Pentatomomorpha | Pentatomoidea | Urostylididae | *Urolabida histrionica* | 2020.7.30 | tropical | humid | Yunnan | 22°8'9''N 99°40'55''E | 940m |
| 20111303 | Pentatomomorpha | Pentatomoidea | Urostylididae | *Urochela quadrinotata* | 2020.10.17 | subtropical | humid | Shaanxi | 33°46′8″N 108°48′23″E | 1334m |
| 20112408 | Pentatomomorpha | Pentatomoidea | Plataspidae | *Coptosoma notabilis* | 2020.8.24 | temperate | sub-humid | Shanxi | 35°33'47''N 112°50'15''E | 1001m |
| 19102504 | Pentatomomorpha | Pentatomoidea | Plataspidae | *Coptosoma* sp. | 2019.8.15 | subtropical | humid | Yunnan | 25°17'42''N 98°47'40''E | 1560m |
| 19100903 | Pentatomomorpha | Pentatomoidea | Plataspidae | *Megacopta cribraria* | 2019.9.18 | subtropical | humid | Guangdong | 24°42'55''N 114°15'37''E | 397m |
| 19102506 | Pentatomomorpha | Pentatomoidea | Plataspidae | *Megacopta cribraria* | 2019.7.20 | tropical | humid | Yunnan | 21°57'58''N 101°12'32''E | 580m |
| 21062416 | Pentatomomorpha | Pentatomoidea | Parastrachiidae | *Parastrachia japonensis* | 2021.6.1 | subtropical | humid | Guangxi | 25°52′11″N 110°25′26″E | 1027m |
| 19092804 | Pentatomomorpha | Pentatomoidea | Acanthosomatidae | *Acanthosoma distinctum* | 2019.9.16 | subtropical | humid | Guangxi | 25°25'18''N 109°9'38''E | 1303m |
| 19102402 | Pentatomomorpha | Pentatomoidea | Acanthosomatidae | *Elasmucha* sp. | 2019.9.6 | subtropical | humid | Yunnan | 23°15'28''N 100°12'24''E | 1792m |
| 19082901 | Pentatomomorpha | Pentatomoidea | Acanthosomatidae | *Elasmucha truncalela* | 2019.7.28 | tropical | humid | Yunnan | 21°51'10''N 100°35'27''E | 1878m |
| 20112415 | Pentatomomorpha | Pentatomoidea | Acanthosomatidae | *Lindbergicoris hochii* | 2020.8.27 | temperate | semi-arid | Ningxia | 35°19'2''N 106°21'3''E | 2191m |
| 19102805 | Pentatomomorpha | Pentatomoidea | Acanthosomatidae | *Microdeuterus megacephalus* | 2019.9.13 | subtropical | humid | Guangxi | 22°11'57''N 106°42'29''E | 100m |
| 19101406 | Pentatomomorpha | Pentatomoidea | Acanthosomatidae | *Microdeuterus spineus* | 2019.8.11 | subtropical | humid | Yunnan | 21°31'42''N 99°4'46''E | 1540m |
| 20112414 | Pentatomomorpha | Pentatomoidea | Acanthosomatidae | *Sastragala esakii* | 2020.8.21 | temperate | sub-humid | Shanxi | 35°14'34''N 112°28'17''E | 564m |
| 19102403 | Pentatomomorpha | Pentatomoidea | Scutelleridae | *Eucorysses grandis* | 2019.8.13 | subtropical | humid | Yunnan | 25°17'42''N 98°47'40''E | 1539m |
| 20112407 | Pentatomomorpha | Pentatomoidea | Scutelleridae | *Hyperoncus lateritius* | 2020.8.18 | subtropical | humid | Tibet | 28°21'25''N 97°1'13''E | 1353m |
| 19100905 | Pentatomomorpha | Pentatomoidea | Scutelleridae | *Lamprocoris roylii* | 2019.8.24 | subtropical | humid | Yunnan | 24°47'7''N 97°40'17''E | 1385m |
| 19092307 | Pentatomomorpha | Pentatomoidea | Scutelleridae | *Poecilocoris latus* | 2019.9.18 | subtropical | humid | Guangdong | 24°42'55''N 114°15'37''E | 397m |
| 19092003 | Pentatomomorpha | Pentatomoidea | Scutelleridae | *Solenosthedium rubropunctatum* | 2019.8.8 | tropical | humid | Yunnan | 23°30'43''N 98°57'0''E | 440m |
| 20112416 | Pentatomomorpha | Pentatomoidea | Cydnidae | *Adomerus notatus* | 2020.8.11 | temperate | semi-arid | Inner Mongolia | 40°50'4''N 111°32'45''E | 1089m |
| 19102206 | Pentatomomorpha | Pentatomoidea | Cydnidae | *Geotomus convexus* | 2019.8.5 | tropical | humid | Yunnan | 23°16'26''N 99°4'26''E | 566m |

continued

| 19102501 | Pentatomomorpha | Pentatomoidea | Cydnidae | *Geotomus convexus* | 2019.8.1 | subtropical | humid | Yunnan | 22°39'45''N 100°22'19''E | 896m |
| --- | --- | --- | --- | --- | --- | --- | --- | --- | --- | --- |
| 19102503 | Pentatomomorpha | Pentatomoidea | Cydnidae | *Macroscyrtus* sp. | 2019.8.1 | subtropical | humid | Yunnan | 22°39'45''N 100°22'19''E | 896m |
| 19102204 | Pentatomomorpha | Pentatomoidea | Tessaratomidae | *Eurostus ochraceus* | 2019.8.16 | subtropical | humid | Yunnan | 25°1'4''N 98°28'43''E | 1896m |
| 19090905 | Pentatomomorpha | Pentatomoidea | Tessaratomidae | *Eusthenes cupreus* | 2019.8.16 | subtropical | humid | Yunnan | 25°1'4'' 98°28'43''E | 1896m |
| 19102203 | Pentatomomorpha | Pentatomoidea | Tessaratomidae | *Pycanum ochraceum* | 2019.7.27 | tropical | humid | Yunnan | 21°51'10''N 100°35'27''E | 1878m |
| 19102201 | Pentatomomorpha | Pentatomoidea | Dinidoridae | *Coridius chinensis* | 2019.7.18 | subtropical | humid | Yunnan | 22°35'1''N 101°1'13''E | 893m |
| 19092704 | Pentatomomorpha | Pentatomoidea | Dinidoridae | *Coridius fuscus* | 2019.9.10 | tropical | humid | Yunnan | 21°29'31''N 101°33'16''E | 637m |
| 19102401 | Pentatomomorpha | Pentatomoidea | Dinidoridae | *Cyclopelta obscura* | 2019.7.23 | tropical | humid | Yunnan | 21°36'1''N 100°41'11''E | 744m |
| 19100902 | Pentatomomorpha | Pentatomoidea | Dinidoridae | *Sagriva banna* | 2019.8.18 | subtropical | humid | Yunnan | 25°13'48''N 98°1'28''E | 1780m |
| 19101405 | Pentatomomorpha | Pentatomoidea | Pentatomidae | *Aeschrocoris obscurus* | 2019.9.4 | subtropical | humid | Yunnan | 24°7'11''N 97°58'52''E | 912m |
| 20070608 | Pentatomomorpha | Pentatomoidea | Pentatomidae | *Axiagastus rosmaus* | 2020.5.8 | tropical | humid | Yunnan | 22°29'63''N 102°15'6''E | 1144m |
| 20112406 | Pentatomomorpha | Pentatomoidea | Pentatomidae | *Brachycerocoris camelus* | 2020.8.22 | temperate | sub-humid | Shanxi | 35°14'34''N 112°28'17''E | 514m |
| 19082902 | Pentatomomorpha | Pentatomoidea | Pentatomidae | *Carbttla crassiventris* | 2019.8.16 | subtropical | humid | Yunnan | 25°1'4''N 98°28'43''E | 1896m |
| 19100901 | Pentatomomorpha | Pentatomoidea | Pentatomidae | *Caystrus obscurus* | 2019.9.14 | subtropical | humid | Guangxi | 22°1'33''N 106°44'44''E | 455m |
| 19102903 | Pentatomomorpha | Pentatomoidea | Pentatomidae | *Caystrus obscurus* | 2019.9.14 | subtropical | humid | Guangxi | 22°1'33''N 106°44'44''E | 450m |
| 19102408 | Pentatomomorpha | Pentatomoidea | Pentatomidae | *Cazira* sp. | 2019.7.28 | tropical | humid | Yunnan | 21°50'14''N 100°35'11''E | 1878m |
| 19102202 | Pentatomomorpha | Pentatomoidea | Pentatomidae | *Chalcopis glandulosa* | 2019.9.13 | subtropical | humid | Guangxi | 22°1'33''N 106°44'44''E | 450m |
| 19103003 | Pentatomomorpha | Pentatomoidea | Pentatomidae | *Cressona* sp. | 2020.7.30 | tropical | humid | Yunnan | 22°8'9''N 99°40'56''E | 940m |
| 19092001 | Pentatomomorpha | Pentatomoidea | Pentatomidae | *Critheus indicus* | 2019.8.12 | subtropical | humid | Yunnan | 25°7'11''N 99°9'4''E | 1758m |
| 19092810 | Pentatomomorpha | Pentatomoidea | Pentatomidae | *Dalpada oculata* | 2019.9.10 | tropical | humid | Yunnan | 21°29'31''N 101°33'16''E | 637m |
| 19102901 | Pentatomomorpha | Pentatomoidea | Pentatomidae | *Dalpada oculata* | 2019.8.28 | subtropical | humid | Yunnan | 23°58'8''N 97°35'21''E | 958m |
| 19102404 | Pentatomomorpha | Pentatomoidea | Pentatomidae | *Dalpada* sp. | 2019.8.12 | subtropical | humid | Yunnan | 25°7'11''N 99°9'4''E | 1758m |
| 19090909 | Pentatomomorpha | Pentatomoidea | Pentatomidae | *Erthesina fullo* | 2019.8.7 | tropical | humid | Yunnan | 25°1'4''N 98°57'47''E | 1320m |
| 19120301 | Pentatomomorpha | Pentatomoidea | Pentatomidae | *Erthesina fullo* | 2019.9.18 | subtropical | humid | Guangdong | 24°42'55''N 114°15'37''E | 397m |
| 19101404 | Pentatomomorpha | Pentatomoidea | Pentatomidae | *Eurydema dominulus* | 2019.9.9 | tropical | humid | Yunnan | 21°15'79''N 101°16'40''E | 633m |
| 19102505 | Pentatomomorpha | Pentatomoidea | Pentatomidae | *Eysarcoris motivagus* | 2019.7.20 | tropical | humid | Yunnan | 21°57'58''N 101°12'33''E | 580m |
| 19102307 | Pentatomomorpha | Pentatomoidea | Pentatomidae | *Eysarcoris ventralis* | 2019.9.29 | subtropical | sub-humid | Tianjin | 38°49'39''N 117°26'53''E | 0m |
| 19092006 | Pentatomomorpha | Pentatomoidea | Pentatomidae | *Gonopsimorpha nigrosignata* | 2019.8.12 | subtropical | humid | Yunnan | 25°7'11''N 99°9'4''E | 1758m |
| 19090911 | Pentatomomorpha | Pentatomoidea | Pentatomidae | *Gonopsis coccinea* | 2019.8.6 | tropical | humid | Yunnan | 23°16'26''N 99°4'26''E | 658m |
| 19102302 | Pentatomomorpha | Pentatomoidea | Pentatomidae | *Halyomorpha halys* | 2019.9.13 | subtropical | humid | Guangxi | 22°11'57''N 106°42'29''E | 100m |
| 19092703 | Pentatomomorpha | Pentatomoidea | Pentatomidae | *Hoplistodera fergussoni* | 2019.9.16 | subtropical | humid | Guangxi | 25°25'18''N 109°9'38''E | 1303m |
| 19092808 | Pentatomomorpha | Pentatomoidea | Pentatomidae | *Hoplistodera pulchra* | 2019.9.16 | subtropical | humid | Guangxi | 25°25'18''N 109°9'38''E | 1303m |
| 19101408 | Pentatomomorpha | Pentatomoidea | Pentatomidae | *Megarrhamphus truncatus* | 2019.9.18 | subtropical | humid | Guangdong | 24°42'55''N 114°15'37''E | 397m |
| 19092807 | Pentatomomorpha | Pentatomoidea | Pentatomidae | *Menida violacea* | 2019.9.16 | subtropical | humid | Guangxi | 25°25'18''N 109°9'38''E | 1303m |
| 20070506 | Pentatomomorpha | Pentatomoidea | Pentatomidae | *Nezara antennata* | 2020.5.7 | subtropical | humid | Yunnan | 22°53'31''N 102°17'46''E | 1780m |
| 20070606 | Pentatomomorpha | Pentatomoidea | Pentatomidae | *Nezara antennata* | 2020.5.7 | subtropical | humid | Yunnan | 22°53'31''N 102°17'46''E | 1780m |
| 19092304 | Pentatomomorpha | Pentatomoidea | Pentatomidae | *Nezara viridula* | 2019.9.1 | subtropical | humid | Yunnan | 24°45'44''N 97°34'56''E | 1191m |
| 20070605 | Pentatomomorpha | Pentatomoidea | Pentatomidae | *Ochrophara chinensis* | 2020.5.22 | subtropical | humid | Guangxi | 21°40'31''N 108°0'4''E | 9m |
| 19090401 | Pentatomomorpha | Pentatomoidea | Pentatomidae | *Plautia fimbriata* | 2019.8.13 | subtropical | humid | Yunnan | 25°17'42''N 98°47'40''E | 1539m |
| 20112405 | Pentatomomorpha | Pentatomoidea | Pentatomidae | *Plautia stali* | 2020.8.22 | temperate | sub-humid | Shanxi | 35°14'34''N 112°28'17''E | 514m |
| 20070601 | Pentatomomorpha | Pentatomoidea | Pentatomidae | *Priassus exemptus* | 2020.5.7 | subtropical | humid | Yunnan | 22°53'31''N 102°17'46''E | 1780m |
| 20070901 | Pentatomomorpha | Pentatomoidea | Pentatomidae | *Scotinophara coarctata* | 2020.5.22 | subtropical | humid | Guangxi | 21°40'31''N 108°0'4''E | 9m |

continued

| 19102205 | Pentatomomorpha | Pentatomoidea | Pentatomidae | *Scotinophara* sp. | 2019.7.16 | subtropical | humid | Guangxi | 22°8'0''N 108°16'10''E | 2m |
| --- | --- | --- | --- | --- | --- | --- | --- | --- | --- | --- |
| 19092005 | Pentatomomorpha | Pentatomoidea | Pentatomidae | *Tolumnia latipes* | 2019.8.9 | tropical | humid | Yunnan | 23°30'43''N 98°57'0''E | 440m |
| 19102407 | Pentatomomorpha | Pentatomoidea | Pentatomidae | *Tolumnia* sp. | 2019.7.29 | tropical | humid | Yunnan | 22°0'38''N 100°14'19''E | 1176m |
| 19090901 | Pentatomomorpha | Pyrrhocoroidea | Pyrrhocoridae | *Antilochus nigripes* | 2019.7.31 | subtropical | humid | Yunnan | 22°39'45''N 100°22'19''E | 1203m |
| 19090406 | Pentatomomorpha | Pyrrhocoroidea | Pyrrhocoridae | *Dysdercus cingulatus* | 2019.8.8 | tropical | humid | Yunnan | 23°30'43''N 98°57'1''E | 440m |
| 20070505 | Pentatomomorpha | Pyrrhocoroidea | Pyrrhocoridae | *Dysdercus fuscomaculatus* | 2020.5.7 | subtropical | humid | Yunnan | 22°53'31''N 102°17'46''E | 1780m |
| 20070607 | Pentatomomorpha | Pyrrhocoroidea | Pyrrhocoridae | *Euscopus fuscus* | 2020.5.7 | tropical | humid | Yunnan | 22°29'62''N 102°15'6''E | 1143m |
| 20112409 | Pentatomomorpha | Pyrrhocoroidea | Pyrrhocoridae | *Pyrrhocoris sinuaticollis* | 2020.8.25 | temperate | sub-humid | Shanxi | 35°29'29''N 112°53'23''E | 761m |
| 20070503 | Pentatomomorpha | Pyrrhocoroidea | Largidae | *Delacampius* sp. | 2020.5.22 | subtropical | humid | Guangxi | 21°40'31''N 108°0'4''E | 9m |
| 19091704 | Pentatomomorpha | Pyrrhocoroidea | Largidae | *Iphita limbata* | 2019.7.19 | tropical | humid | Yunnan | 21°56'6''N 101°15'6''E | 893m |
| 19092305 | Pentatomomorpha | Pyrrhocoroidea | Largidae | *Iphita limbata* | 2019.8.26 | subtropical | humid | Yunnan | 24°45'44''N 97°34'56''E | 620m |
| 19090912 | Pentatomomorpha | Pyrrhocoroidea | Largidae | *Macroceroea grandis* | 2019.7.20 | tropical | humid | Yunnan | 21°56'6''N 101°15'6 ''E | 569m |
| 19082905 | Pentatomomorpha | Pyrrhocoroidea | Largidae | *Physopelta gutta* | 2019.7.16 | subtropical | humid | Guangxi | 22°8'1''N 108°16'10''E | 2m |
| 19102406 | Pentatomomorpha | Pyrrhocoroidea | Largidae | *Physopelta quadriguttata* | 2019.7.28 | tropical | humid | Yunnan | 21°50'14''N 100°35'11''E | 1878m |
| 21012803 | Pentatomomorpha | Coreoidea | Stenocephalidae | *Dicranocephalus* sp. | 2015.7.19 | temperate | humid | Budapest | 47°29′33″N 19°03′05″E | 365m |
| 20112410 | Pentatomomorpha | Coreoidea | Rhopalidae | *Chorosoma gracile* | 2020.8.11 | temperate | semi-arid | Inner Mongolia | 40°50'4''N 111°32'45''E | 1089m |
| 20112412 | Pentatomomorpha | Coreoidea | Rhopalidae | *Corizus tetraspilus* | 2020.8.4 | temperate | semi-arid | Inner Mongolia | 40°50'4''N 111°32'45''E | 1089m |
| 20070406 | Pentatomomorpha | Coreoidea | Rhopalidae | *leptocoris vicinus* | 2020.7.2 | subtropical | humid | Guangdong | 23°5'45''N 113°17'25''E | 4m |
| 20112411 | Pentatomomorpha | Coreoidea | Rhopalidae | *Stictopleurus abutilon* | 2020.8.4 | temperate | arid | Inner Mongolia | 41°57'8''N 101°5'10''E | 888m |
| 20112420 | Pentatomomorpha | Coreoidea | Alydidae | *Alydus zichyi* | 2020.8.11 | temperate | semi-arid | Inner Mongolia | 40°50'4''N 111°32'45''E | 1089m |
| 20070902 | Pentatomomorpha | Coreoidea | Alydidae | *Grypocephalus pallipectus* | 2020.5.20 | subtropical | humid | Guangxi | 23°0'42''N 105°47'28''E | 225m |
| 19100904 | Pentatomomorpha | Coreoidea | Alydidae | *Leptocorisa lepida* | 2019.9.13 | subtropical | humid | Guangxi | 22°11'57''N 106°42'29''E | 100m |
| 19102107 | Pentatomomorpha | Coreoidea | Alydidae | *Leptocorisa lepida* | 2019.8.22 | subtropical | humid | Yunnan | 25°2'29''N 97°43'37''E | 600m |
| 19102102 | Pentatomomorpha | Coreoidea | Alydidae | *Leptocorisa oratoria* | 2019.8.22 | subtropical | humid | Yunnan | 25°2'29''N 97°43'37''E | 600m |
| 20112419 | Pentatomomorpha | Coreoidea | Alydidae | *Megalotomus junceus* | 2020.8.24 | temperate | sub-humid | Shanxi | 35°33'47''N 112°50'15''E | 1001m |
| 19100907 | Pentatomomorpha | Coreoidea | Alydidae | *Riptortus linearis* | 2019.9.9 | tropical | humid | Yunnan | 21°15'27''N 101°16'40''E | 633m |
| 19102103 | Pentatomomorpha | Coreoidea | Alydidae | *Riptortus linearis* | 2019.9.13 | subtropical | humid | Guangxi | 22°11'57''N 106°42'29''E | 100m |
| 19092302 | Pentatomomorpha | Coreoidea | Alydidae | *Riptortus pedestris* | 2019.9.18 | subtropical | humid | Guangdong | 24°42'55''N 114°15'37''E | 397m |
| 19090906 | Pentatomomorpha | Coreoidea | Coreidae | *Acanthocoris scaber* | 2019.7.29 | subtropical | humid | Yunnan | 22°0'38''N 100°14'19''E | 1176m |
| 19091703 | Pentatomomorpha | Coreoidea | Coreidae | *Cletomorpha raja* | 2019.8.17 | subtropical | humid | Yunnan | 25°1'4''N 98°28'43''E | 1896m |
| 19102108 | Pentatomomorpha | Coreoidea | Coreidae | *Cletus trigonus* | 2019.7.21 | tropical | humid | Yunnan | 21°57'56''N 101°12'33''E | 580m |
| 19102902 | Pentatomomorpha | Coreoidea | Coreidae | *Cletus trigonus* | 2019.8.9 | tropical | humid | Yunnan | 23°30'43''N 98°57'0''E | 440m |
| 19102906 | Pentatomomorpha | Coreoidea | Coreidae | *Cloresmus. modestus* | 2019.9.19 | tropical | humid | Yunnan | 21°56'6''N 101°15'6''E | 569m |
| 19092701 | Pentatomomorpha | Coreoidea | Coreidae | *Cloresmus modestus* | 2019.9.10 | tropical | humid | Yunnan | 21°29'31''N 101°33'16''E | 637m |
| 19090405 | Pentatomomorpha | Coreoidea | Coreidae | *Cloreunus modestus* | 2019.8.5 | tropical | humid | Yunnan | 23°16'26''N 99°4'26''E | 566m |
| 19091712 | Pentatomomorpha | Coreoidea | Coreidae | *Derepteryx hardwickii* | 2019.8.1 | subtropical | humid | Yunnan | 22°39'45''N 100°22'19''E | 896m |
| 19092809 | Pentatomomorpha | Coreoidea | Coreidae | *Homoeocerus bipunctatus* | 2019.9.10 | tropical | humid | Yunnan | 21°29'31''N 101°33'16''E | 637m |
| 19102804 | Pentatomomorpha | Coreoidea | Coreidae | *Homoeocerus bipustulatus* | 2020.7.30 | tropical | humid | Yunnan | 22°8'10''N 99°40'56''E | 940m |
| 19102105 | Pentatomomorpha | Coreoidea | Coreidae | *Homoeocerus striicornis* | 2019.9.13 | subtropical | humid | Guangxi | 22°11'57''N 106°42'29''E | 100m |
| 19102301 | Pentatomomorpha | Coreoidea | Coreidae | *Homoeocerus unipunctatus* | 2019.9.13 | subtropical | humid | Guangxi | 22°11'57''N 106°42'29''E | 100m |
| 19102106 | Pentatomomorpha | Coreoidea | Coreidae | *Hydaropsis longirostris* | 2019.8.6 | tropical | humid | Yunnan | 23°15'25''N 99°3'42''E | 658m |
| 19102802 | Pentatomomorpha | Coreoidea | Coreidae | *Hydaropsis longirostris* | 2019.9.3 | subtropical | humid | Yunnan | 24°5'23''N 98°4'14''E | 880m |

continued

| 19092812 | Pentatomomorpha | Coreoidea | Coreidae | *Hygia touchei* | 2019.9.16 | subtropical | humid | Guangxi | 25°25'18''N 109°9'38''E | 1303m |
| --- | --- | --- | --- | --- | --- | --- | --- | --- | --- | --- |
| 20112417 | Pentatomomorpha | Coreoidea | Coreidae | *Hygia touchei* | 2020.8.17 | subtropical | humid | Shaanxi | 33°46'8''N 108°48'23''E | 1334m |
| 20112418 | Pentatomomorpha | Coreoidea | Coreidae | *Mygdonia* sp. | 2020.10.11 | subtropical | humid | Shaanxi | 33°46'37''N 108°45'4''E | 1291m |
| 19092002 | Pentatomomorpha | Coreoidea | Coreidae | *Notobitus excellens* | 2019.8.12 | subtropical | humid | Yunnan | 25°7'11''N 99°9'4''E | 1758m |
| 19102104 | Pentatomomorpha | Coreoidea | Coreidae | *Notobitus* sp. | 2019.7.21 | tropical | humid | Yunnan | 21°57'58''N 101°12'33''E | 580m |
| 19090908 | Pentatomomorpha | Coreoidea | Coreidae | *Ochrochira ferruginea* | 2019.7.28 | tropical | humid | Yunnan | 21°51'10''N 100°35'27''E | 1613m |
| 19092705 | Pentatomomorpha | Coreoidea | Coreidae | *Paradasynus* sp. | 2019.8.13 | subtropical | humid | Yunnan | 25°17'42''N 98°47'40''E | 1539m |
| 19092004 | Pentatomomorpha | Coreoidea | Coreidae | *Physomerus grossipes* | 2019.8.8 | tropical | humid | Yunnan | 23°30'43''N 98°57'0 ''E | 440m |
| 19102304 | Pentatomomorpha | Lygaeoidea | Pachygronthidae | *Pachygrontha lurida* | 2019.9.1 | subtropical | humid | Yunnan | 24°4'25''N 97°48'49''E | 1190m |
| 19120209 | Pentatomomorpha | Lygaeoidea | Pachygronthidae | *Pachyphlelgyas modiglianii* | 2019.9.3 | subtropical | humid | Yunnan | 24°5'23''N 98°4'14''E | 880m |
| 20111507 | Pentatomomorpha | Lygaeoidea | Pachygronthidae | *Pachygrontha antennata* | 2020.8.22 | temperate | sub-humid | Shanxi | 35°14'34''N 112°28'17''E | 564m |
| 19091711 | Pentatomomorpha | Lygaeoidea | Heterogastridae | *Dinomachus sikhimensis* | 2019.7.22 | tropical | humid | Yunnan | 21°57'58''N 101°12'33''E | 580m |
| 19120202 | Pentatomomorpha | Lygaeoidea | Heterogastridae | *Heterogaster* sp. | 2019.8.11 | subtropical | humid | Yunnan | 21°31'42''N 99°4'46''E | 1540m |
| 20070502 | Pentatomomorpha | Lygaeoidea | Heterogastridae | *Nerthus* sp. | 2020.5.6 | tropical | humid | Yunnan | 22°29'61''N 102°15'6''E | 1142m |
| 20111514 | Pentatomomorpha | Lygaeoidea | Heterogastridae | *Nerthus taivanicus* | 2020.10.11 | subtropical | humid | Shaanxi | 33°46'37''N 108°45'4''E | 1291m |
| 19120201 | Pentatomomorpha | Lygaeoidea | Heterogastridae | *Sadoletus bakeri* | 2019.7.31 | subtropical | humid | Yunnan | 22°39'45''N 100°22'19''E | 896m |
| 19120210 | Pentatomomorpha | Lygaeoidea | Artheneidae | *Artheneis intricata* | 2019.9.29 | subtropical | sub-humid | Tianjin | 38°49'39''N 117°26'53''E | 0m |
| 19101803 | Pentatomomorpha | Lygaeoidea | Berytidae | *Metacanthus Pulchellus* | 2019.7.20 | subtropical | humid | Yunnan | 22°35'1''N 101°1'14''E | 897m |
| 19102904 | Pentatomomorpha | Lygaeoidea | Berytidae | *Metatropis brevirostris* | 2018.8.28 | subtropical | humid | Yunnan | 23°58'8''N 97°35'21''E | 950m |
| 20111515 | Pentatomomorpha | Lygaeoidea | Berytidae | *Paraberytus* sp. | 2020.9.3 | subtropical | humid | Yunnan | 24°26'52''N 97°34'43''E | 380m |
| 19101806 | Pentatomomorpha | Lygaeoidea | Berytidae | *Yemmalysus* sp. | 2019.9.13 | subtropical | humid | Yunnan | 22°7'14''N 106°44'13''E | 253m |
| 19102905 | Pentatomomorpha | Lygaeoidea | Berytidae | *Yemmatropis* sp. | 2019.8.17 | subtropical | humid | Yunnan | 25°1'4''N 98°28'43''E | 1898m |
| 19102803 | Pentatomomorpha | Lygaeoidea | Rhyparochromidae | *Appolonius crassus* | 2019.7.25 | tropical | humid | Yunnan | 21°34'36''N 100°18'27''E | 1073m |
| 19091708 | Pentatomomorpha | Lygaeoidea | Rhyparochromidae | *Botocudo marginatus* | 2019.7.24 | tropical | humid | Yunnan | 21°34'36''N 100°18'27''E | 1073m |
| 19103001 | Pentatomomorpha | Lygaeoidea | Rhyparochromidae | *Botocudo marianensis* | 2019.8.24 | subtropical | humid | Yunnan | 25°2'29''N 97°43'37''E | 600m |
| 19090907 | Pentatomomorpha | Lygaeoidea | Rhyparochromidae | *Caridops pseudadmistus* | 2019.8.12 | subtropical | humid | Yunnan | 25°7'11''N 99°9'4''E | 1758m |
| 19092811 | Pentatomomorpha | Lygaeoidea | Rhyparochromidae | *Elasmdomus squalidus* | 2019.8.8 | tropical | humid | Yunnan | 23°30'43''N 98°57'0''E | 440m |
| 19103004 | Pentatomomorpha | Lygaeoidea | Rhyparochromidae | *Elasmolomus squalidus* | 2019.8.1 | subtropical | humid | Yunnan | 22°39'45''N 100°22'19''E | 896m |
| 19092706 | Pentatomomorpha | Lygaeoidea | Rhyparochromidae | *Gastrodes chinesis* | 2019.9.16 | subtropical | humid | Guangxi | 25°25'18''N 109°9'38''E | 1303m |
| 20111305 | Pentatomomorpha | Lygaeoidea | Rhyparochromidae | *Gastrodes grossipes* | 2020.8.1 | temperate | arid | Ningxia | 38°44'55''N 105°54'59''E | 1937m |
| 19091701 | Pentatomomorpha | Lygaeoidea | Rhyparochromidae | *Horridipamera lateralis* | 2019.7.16 | subtropical | humid | Guangxi | 22°8'0''N 108°16'10''E | 2m |
| 19101808 | Pentatomomorpha | Lygaeoidea | Rhyparochromidae | *Metochus thoracicus* | 2019.7.20 | subtropical | humid | Yunnan | 21°56'6''N 101°15'6''E | 569m |
| 19092306 | Pentatomomorpha | Lygaeoidea | Rhyparochromidae | *Pamerarma punctulata* | 2019.8.26 | subtropical | humid | Yunnan | 24°45'44''N 97°34'56''E | 620m |
| 20112421 | Pentatomomorpha | Lygaeoidea | Rhyparochromidae | *Plinthisus* sp. | 2020.10.11 | subtropical | humid | Shaanxi | 33°46'37''N 108°45'4''E | 1291m |
| 19092308 | Pentatomomorpha | Lygaeoidea | Rhyparochromidae | *Primierus longispinus* | 2019.9.16 | subtropical | humid | Guangxi | 25°25'18''N 109°9'38''E | 1303m |
| 19091702 | Pentatomomorpha | Lygaeoidea | Rhyparochromidae | *Rhyparothesus dudgeoni* | 2019.7.31 | subtropical | humid | Yunnan | 22°39'45''N 100°22'19''E | 896m |
| 19092309 | Pentatomomorpha | Lygaeoidea | Colobathristidae | *Phaenacantha bicolor* | 2019.9.18 | subtropical | humid | Guangdong | 24°42'5''N 114°15'37''E | 397m |
| 20111502 | Pentatomomorpha | Lygaeoidea | Geocoridae | *Engistus salinus* | 2020.8.8 | temperate | arid | Inner Mongolia | 40°49'43''N 108°46'9''E | 996m |
| 20112424 | Pentatomomorpha | Lygaeoidea | Geocoridae | *Geocoris dispar* | 2020.7.28 | temperate | semi-arid | Ningxia | 35°33'32''N 106°25'50''E | 1762m |
| 20111504 | Pentatomomorpha | Lygaeoidea | Geocoridae | *Geocoris flaviceps* | 2020.8.21 | temperate | sub-humid | Shanxi | 35°16'25''N 112°26'27''E | 658m |
| 20111516 | Pentatomomorpha | Lygaeoidea | Geocoridae | *Geocoris grylloides* | 2020.8.11 | temperate | semi-arid | Inner Mongolia | 40°50'4''N 111°32'45''E | 1089m |
| 19102508 | Pentatomomorpha | Lygaeoidea | Geocoridae | *Geocoris* sp. | 2019.9.2 | subtropical | humid | Yunnan | 24°5'59''N 97°55'59''E | 950m |

continued

| 20112422 | Pentatomomorpha | Lygaeoidea | Geocoridae | *Henestaris oschanini* | 2020.8.8 | temperate | arid | Inner Mongolia | 40°49'43''N 108°46'9''E | 996m |
| --- | --- | --- | --- | --- | --- | --- | --- | --- | --- | --- |
| 20111304 | Pentatomomorpha | Lygaeoidea | Lygaeidae | *Caenocoris* sp. | 2020.10.17 | subtropical | humid | Shaanxi | 33°45'46''N 108°42'59''E | 1074m |
| 20111511 | Pentatomomorpha | Lygaeoidea | Lygaeidae | *Lygaeus hanseni* | 2020.8.1 | temperate | arid | Ningxia | 38°44'56''N 105°54'59''E | 1937m |
| 20112423 | Pentatomomorpha | Lygaeoidea | Lygaeidae | *Nysius eximius* | 2020.8.8 | temperate | arid | Inner Mongolia | 40°49'43''N 108°46'9''E | 996m |
| 20111512 | Pentatomomorpha | Lygaeoidea | Lygaeidae | *Nysius nigriconis* | 2020.8.4 | temperate | arid | Inner Mongolia | 41°57'8''N 101°5'10''E | 888m |
| 20111505 | Pentatomomorpha | Lygaeoidea | Lygaeidae | *Pylorgus sordidus* | 2020.8.15 | subtropical | humid | Shaanxi | 33°46'8''N 108°48'23''E | 1334m |
| 20111510 | Pentatomomorpha | Lygaeoidea | Lygaeidae | *Pylorgus sordidus* | 2020.8.15 | subtropical | humid | Shaanxi | 33°46'8''N 108°48'23''E | 1334m |
| 20070504 | Pentatomomorpha | Lygaeoidea | Lygaeidae | *Spilostethus hospes* | 2020.5.25 | tropical | humid | Hainan | 18°42'35''N 109°50'28''E | 570m |
| 19120208 | Pentatomomorpha | Lygaeoidea | Malcidae | *Malcus auriculatus* | 2019.8.15 | subtropical | humid | Yunnan | 25°17'42''N 98°47'40''E | 1560m |
| 19120207 | Pentatomomorpha | Lygaeoidea | Malcidae | *Malcus* cf. *arcuatus* | 2019.8.29 | subtropical | humid | Yunnan | 23°59'6''N 97°36'43''E | 850m |
| 19090402 | Pentatomomorpha | Lygaeoidea | Malcidae | *Malcus elongatus* | 2019.8.7 | tropical | humid | Yunnan | 25°1'4''N 98°57'47''E | 1320m |
| 19090403 | Pentatomomorpha | Lygaeoidea | Malcidae | *Malcus flavidipes* | 2019.8.8 | tropical | humid | Yunnan | 23°30'43''N 98°57'1''E | 440m |
| 19102308 | Pentatomomorpha | Lygaeoidea | Malcidae | *Malcus flavidipes* | 2019.7.21 | tropical | humid | Yunnan | 21°57'58''N 101°12'32''E | 580m |
| 19101801 | Pentatomomorpha | Lygaeoidea | Malcidae | *Malcus furcatus* | 2019.8.18 | subtropical | humid | Yunnan | 25°13'48''N 98°1'28''E | 1780m |
| 19090404 | Pentatomomorpha | Lygaeoidea | Malcidae | *Malcus noduliferus* | 2019.8.17 | subtropical | humid | Yunnan | 25°1'4''N 98°28'43''E | 1896m |
| 19092301 | Pentatomomorpha | Lygaeoidea | Malcidae | *Malcus setosus* | 2019.8.28 | subtropical | humid | Yunnan | 23°58'8''N 97°35'21''E | 950m |
| 19091705 | Pentatomomorpha | Lygaeoidea | Malcidae | *Malcus sinicus* | 2019.8.15 | subtropical | humid | Yunnan | 25°17'42''N 98°47'40''E | 1560m |
| 19101805 | Pentatomomorpha | Lygaeoidea | Malcidae | *Malcus*.cf.*indicus* | 2019.8.22 | subtropical | humid | Yunnan | 25°2'29''N 97°43'37''E | 600m |

**Supplementary Table S2. The primers used in this study.**

| Target | Target gene | Primer name | Primer sequence (5'→3') | Length (bp) | Fwd./Rev. | Annealing (℃) | Target length |
| --- | --- | --- | --- | --- | --- | --- | --- |
| Symbionts | 16S rRNA | 27F | AGRGTTTGATYNTGGCTCAG | 20 | Fwd. | 54 | 1500 |
|  | 16S rRNA | 1492R | TASGGHTACCTTGTTASGACTT | 22 | Rev. | 54 |  |
|  | ITS | ITS1 | CTTGGTCATTTAGAGGAAGTAA | 22 | Fwd. | 55 | 650 |
|  | ITS | ITS4 | TCCTCCGCTTATTGATATGC | 20 | Rev. | 55 |  |
| Hosts | 18S rRNA | 18S-Ns1-F | GTAGTCATATGCTTGTCTC | 19 | Fwd. | 53 | 1800 |
|  | 18S rRNA | 18S-Ns8-R | TCCGCAGGTTCACCTACGGA | 20 | Rev. | 53 |  |
|  | 28S rRNA | 28S-SS7-AF | GCGGAGGAAAAGAAACTAAC | 20 | Fwd. | 53 | 1100 |
|  | 28S rRNA | 28S-XS2-AR | GGCATAGTTCACCATCTTTCG | 21 | Rev. | 53 |  |
|  | 28S rRNA | 28S-DF1-BF | ATCCGACCCGTCTTGAAACAC | 21 | Fwd. | 55 | 850 |
|  | 28S rRNA | 28S-FD1-BR | TGCTACTACCACCAAGATCTG | 21 | Rev. | 55 |  |
|  | 28S rRNA | 28S-EE-CF | ATCCGCTAAGGAGTGTGTAA | 20 | Fwd. | 53 | 850 |
|  | 28S rRNA | 28S-GG-CR | CCGACTTCCCTTACCTACATT | 21 | Rev. | 53 |  |
|  | 28S rRNA | 28S-OP-DF | TAGGAGGGAGATAGGGTTTG | 20 | Fwd. | 55 | 1100 |
|  | 28S rRNA | 28S-Pob-DR | TACCGCCCCAGTCAAACTCC | 20 | Rev. | 55 |  |
|  | 28S rRNA | 28S-UZ-EF | AGGTGTAGCATAAGTGGGAG | 20 | Fwd. | 55 | 850 |
|  | 28S rRNA | 28S-ZU-ER | TTCGGTCTTAGAGGCGTTCAG | 21 | Rev. | 55 |  |
|  | 28S rRNA | 28S-cb4-FF | CGGCTCTTCCTATCATTGCG | 20 | Fwd. | 55 | 600 |
|  | 28S rRNA | 28S-kb4-FR | CAGCGTGGCAACTGCTCTCC | 20 | Rev. | 55 |  |
|  | CO1 | 1490 | GGTCAACAAATCATAAAGATATTGG | 25 | Fwd. | 53 | 750 |
|  | CO1 | 2198 | TAAACTTCAGGGTGACCAAAAAATCA | 26 | Rev. | 53 |  |
|  | CO1 | C1-J1709 | AATTGGWGGWTTYGGAAAYTG | 21 | Fwd. | 51 | 1000 |
|  | CO1 | C1-N2776 | GGTAATCAGAGTATCGWCGNGG | 22 | Rev. | 51 |  |
|  | CO2 | J3043 | GGCAGATTAGTGYAATGRATTTAA | 24 | Fwd. | 47 | 700 |
|  | CO2 | N3796 | ACTATTAGATGGTTTAAGAG | 20 | Rev. | 47 |  |

**Supplementary Table S3. The results of PERMANOVA test of the symbiotic bacterial communities in different insect superfamilies.**

|  | Cimicoidea | Naboidea | Miroidea | Reduvioidea | Aradoidea | Pentatomoidea | Pyrrhocoroidea | Coreoidea | Lygaeoidea |
| --- | --- | --- | --- | --- | --- | --- | --- | --- | --- |
| Cimicoidea |  |  |  |  |  |  |  |  |  |
| Naboidea | 0.248 |  |  |  |  |  |  |  |  |
| Miroidea | 0.032 | 0.128 |  |  |  |  |  |  |  |
| Reduvioidea | 0.215 | 0.062 | 0.002 |  |  |  |  |  |  |
| Aradoidea | 0.189 | 0.222 | 0.073 | 0.107 |  |  |  |  |  |
| Pentatomoidea | 0.002 | 0.002 | 0.002 | 0.297 | 0.01 |  |  |  |  |
| Pyrrhocoroidea | 0.044 | 0.03 | 0.002 | 0.002 | 0.043 | 0.002 |  |  |  |
| Coreoidea | 0.002 | 0.002 | 0.002 | 0.002 | 0.006 | 0.002 | 0.002 |  |  |
| Lygaeoidea | 0.006 | 0.011 | 0.005 | 0.006 | 0.049 | 0.002 | 0.002 | 0.007 |  |

The names of superfamily are listed according to the phylogenetic relationship.

**Supplementary Table S4. The results of PERMANOVA test of the symbiotic bacterial communities in different sample sites.**

|  | Guangdong | Guangxi | Hainan | Tianjin | Yunnan | Sichuan | Tibet | Shaanxi | Shanxi | Inner Mongolia | Ningxia |
| --- | --- | --- | --- | --- | --- | --- | --- | --- | --- | --- | --- |
| Guangdong |  |  |  |  |  |  |  |  |  |  |  |
| Guangxi | 0.177 |  |  |  |  |  |  |  |  |  |  |
| Hainan | 0.38 | 0.066 |  |  |  |  |  |  |  |  |  |
| Tianjin | 0.896 | 0.606 | 0.547 |  |  |  |  |  |  |  |  |
| Yunnan | 0.461 | 0.079 | 0.11 | 0.944 |  |  |  |  |  |  |  |
| Sichuan | 0.258 | 0.066 | 0.427 | 0.756 | 0.177 |  |  |  |  |  |  |
| Tibet | 0.944 | 0.309 | 0.309 | 0.819 | 0.519 | 0.536 |  |  |  |  |  |
| Shaanxi | 0.461 | 0.427 | 0.177 | 0.944 | 0.863 | 0.461 | 0.547 |  |  |  |  |
| Shanxi | 0.777 | 0.709 | 0.177 | 0.944 | 0.589 | 0.309 | 0.724 | 0.815 |  |  |  |
| Inner Mongolia | 0.177 | 0.055 | 0.073 | 0.944 | 0.066 | 0.606 | 0.944 | 0.461 | 0.235 |  |  |
| Ningxia | 0.309 | 0.055 | 0.309 | 1 | 0.11 | 0.766 | 0.724 | 0.605 | 0.378 | 0.461 |  |

The names of sample province are listed according to their relative location.

**Supplementary Table S5. The results of PERMANOVA test of the symbiotic fungal communities in different insect superfamilies.**

|  | Cimicoidea | Naboidea | Miroidea | Reduvioidea | Aradoidea | Pentatomoidea | Pyrrhocoroidea | Coreoidea | Lygaeoidea |
| --- | --- | --- | --- | --- | --- | --- | --- | --- | --- |
| Cimicoidea |  |  |  |  |  |  |  |  |  |
| Naboidea | 0.317 |  |  |  |  |  |  |  |  |
| Miroidea | 0.773 | 0.34 |  |  |  |  |  |  |  |
| Reduvioidea | 0.198 | 0.548 | 0.548 |  |  |  |  |  |  |
| Aradoidea | 0.048 | 0.084 | 0.048 | 0.198 |  |  |  |  |  |
| Pentatomoidea | 0.152 | 0.629 | 0.048 | 0.34 | 0.154 |  |  |  |  |
| Pyrrhocoroidea | 0.048 | 0.136 | 0.136 | 0.068 | 0.122 | 0.123 |  |  |  |
| Coreoidea | 0.152 | 0.617 | 0.057 | 0.327 | 0.177 | 0.583 | 0.177 |  |  |
| Lygaeoidea | 0.136 | 0.455 | 0.048 | 0.136 | 0.068 | 0.317 | 0.154 | 0.343 |  |

The names of superfamily are listed according to the phylogenetic relationship.

**Supplementary Table S6. The results of PERMANOVA test of the symbiotic fungal communities in different sample sites.**

|  | Guangdong | Guangxi | Hainan | Tianjin | Yunnan | Sichuan | Tibet | Shaanxi | Shanxi | Inner Mongolia | Ningxia |
| --- | --- | --- | --- | --- | --- | --- | --- | --- | --- | --- | --- |
| Guangdong |  |  |  |  |  |  |  |  |  |  |  |
| Guangxi | 0.175 |  |  |  |  |  |  |  |  |  |  |
| Hainan | 0.46 | 0.615 |  |  |  |  |  |  |  |  |  |
| Tianjin | 0.138 | 0.02 | 0.109 |  |  |  |  |  |  |  |  |
| Yunnan | 0.106 | 0.511 | 0.465 | 0.078 |  |  |  |  |  |  |  |
| Sichuan | 0.359 | 0.287 | 0.418 | 0.094 | 0.223 |  |  |  |  |  |  |
| Tibet | 0.092 | 0.112 | 0.312 | 0.102 | 0.115 | 0.102 |  |  |  |  |  |
| Shaanxi | 0.034 | 0.042 | 0.352 | 0.018 | 0.036 | 0.006 | 0.785 |  |  |  |  |
| Shanxi | 0.002 | 0.002 | 0.247 | 0.003 | 0.001 | 0.01 | 0.638 | 0.339 |  |  |  |
| Inner Mongolia | 0.001 | 0.001 | 0.248 | 0.028 | 0.001 | 0.008 | 0.069 | 0.099 | 0.004 |  |  |
| Ningxia | 0.005 | 0.002 | 0.518 | 0.015 | 0.004 | 0.016 | 0.225 | 0.604 | 0.019 | 0.103 |  |

The names of sample province are listed according to their relative location.

**Supplementary Table S7. The fossil calibrations used in the divergence time estimation.**

| Calibration node | Fossil record | Taxonomic group | Prior distributions (Ma) |
| --- | --- | --- | --- |
| Naboidea | *Karanabis kiritshenkoi* | Naboidea: Nabidae | 166.1–157.3 |
| Tingidae | *Golmonia pater* | Miroidea: Tingidae: Cantacaderinae: Golmoniini | 125.0–113.0 |
| Mirinae | *Cretamystilus herczeki* | Miroidea: Miridae: Mirinae: Mecistoscelini | 99.6–93.5 |
| Mezirinae | *Myanmezira longicornis* | Aradoidea: Aradidae: Mezirinae | 99.6–93.5 |
| Urostylididae | *Urochela pardalina* | Pentatomoidea: Urostylididae: Urostylidinae: Urostylidini | 20.44–15.97 |
| Cydnidae+  (Dinidoridae+Tessaratomidae) | *Cilicydnus robustispinus* | Pentatomoidea: Cydnidae: Amnestinae | 125.45–122.46 |
| Dinidoridae | *Dinidorites margiformis* | Pentatomoidea: Dinidoridae | 56.0–47.8 |
| Acanthosomatidae | *Acanthosoma* sp*.* | Pentatomoidea: Acanthosomatidae | 15.97–11.608 |
| Micrelytrinae | *Eothes elegans* | Coreoidea: Alydidae: Micrelytrinae | 37.2–33.9 |
| Berytidae | *Metacanthus* *serratus* | Lygaeoidea: Berytidae | 33.9–28.4 |

**Supplementary Table S8.** **The insect host families and their dominant symbionts.**

| Host taxon | | Dominant bacterial symbiont | | Symbiotic localization | Transmission modality |
| --- | --- | --- | --- | --- | --- |
| Superfamily | Family | Class | Genus |  |  |
| Reduvioidea | Reduviidae | - | - | - | Absence of dominant symbiont |
| Miroidea | Tingidae | Alphaproteobacteria | *Wolbachia* | Bacteriocyte | Strict vertical transmission |
|  | Miridae | Alphaproteobacteria | *Wolbachia* | Bacteriocyte | Strict vertical transmission |
| Naboidea | Nabidae | Mollicutes  Alphaproteobacteria | *Spiroplasma*  *Wolbachia* | Supposed midgut  Bacteriocyte | Supposed strict vertical transmission |
| Cimicoidea | Anthocoridae | - | - | - | Absence of dominant symbiont |
|  | Plokiophilidae | Mollicutes | *Spiroplasma* | Supposed midgut | Strict vertical transmission |
| Aradoidea | Aradidae | Alphaproteobacteria | *Sphingomonas*  *Wolbachia* | Supposed midgut  Supposed Bacteriocyte | Supposed strict vertical transmission |
| Pentatomoidea | Urostylididae | Gammaproteobacteria | Morganellaceae sp.  *Candidatus* Tachikawaea | Midgut crypts | Non-strict vertical transmission |
|  | Saileriolidae | Gammaproteobacteria | *Erwinia* | Supposed midgut crypts | Supposed non-strict vertical transmission |
|  | Plataspidae | Gammaproteobacteria | *Candidatus* Ishikawaella | Midgut crypts | Non-strict vertical transmission |
|  | Parastrachiidae | Gammaproteobacteria | *Candidatus* Benitsuchiphilus | Midgut crypts | Non-strict vertical transmission |
|  | Acanthosomatidae | Gammaproteobacteria | *Candidatus* Rosenkranzia | Midgut crypts | Non-strict vertical transmission |
|  | Scutelleridae | Gammaproteobacteria | *Pantoea* | Midgut crypts | Non-strict vertical transmission |
|  | Cydnidae | Gammaproteobacteria | *Pantoea* | Midgut crypts | Non-strict vertical transmission |
|  | Tessaratomidae | Gammaproteobacteria | *Pantoea* | Midgut crypts | Non-strict vertical transmission |
|  | Dinidoridae | Gammaproteobacteria | *Pantoea* | Midgut crypts | Non-strict vertical transmission |
|  | Pentatomidae | Gammaproteobacteria | *Pantoea* | Midgut crypts | Non-strict vertical transmission |
| Pyrrhocoroidea | Pyrrhocoridae | Coriobacteriia | *Coriobacterium*  *Gordonibacter* | Midgut M3 region | Non-strict vertical transmission |
|  | Largidae | Betaproteobacteria | *Paraburkholderia* | Midgut crypts | Symbiont-mediated morphogenesis |
| Coreoidea | Stenocephalidae | - | - | - | Unknow |
|  | Rhopalidae | - | - | - | Absence of dominant symbiont |
|  | Alydidae | Betaproteobacteria | *Caballeronia* | Midgut crypts | Symbiont-mediated morphogenesis |
|  | Coreidae | Betaproteobacteria | *Caballeronia* | Midgut crypts | Symbiont-mediated morphogenesis |
| Lygaeoidea | Pachygronthidae | Betaproteobacteria | *Caballeronia* | Supposed midgut crypts | Supposed symbiont-mediated morphogenesis |
|  | Heterogastridae | Betaproteobacteria | *Caballeronia* | Supposed midgut crypts | Supposed symbiont-mediated morphogenesis |
|  | Artheneidae | Gammaproteobacteria | *Pectobacterium* | Bacteriome | Vertical transmission |
|  | Berytidae | Betaproteobacteria | *Caballeronia* | Midgut crypts | Symbiont-mediated morphogenesis |
|  | Rhyparochromidae | Betaproteobacteria | *Caballeronia* | Midgut crypts | Symbiont-mediated morphogenesis |
|  | Colobathristidae | Betaproteobacteria | *Caballeronia* | Supposed midgut crypts | Supposed symbiont-mediated morphogenesis |
|  | Geocoridae | Alphaproteobacteria  Gammaproteobacteria | *Wolbachia*  *Sodalis* | Bacteriome | Strict vertical transmission |
|  | Lygaeidae | Alphaproteobacteria  Gammaproteobacteria | *Wolbachia*  *Schneideria*  *Sodalis* | Bacteriome | Strict vertical transmission |
|  | Malcidae | Alphaproteobacteria | *Wolbachia* | Bacteriome | Strict vertical transmission |
